# Improving High‐Temperature Operational Stability of Inverted Perovskite Solar Cells Through MXene Heat‐Dissipating Layers

**DOI:** 10.1002/advs.77661

**Published:** 2026-09-27

**Authors:** Masoud Karimipour, Nil Monrós Oliveras, Zhenchuan Tian, Timm Swoboda, Francesco Salutari, Marianna Sledzinska, Maria Chiara Spadaro, Kenedy Tabah Tanko, Javier Rodríguez‐Viejo, Jordi Arbiol, Tiankai Zhang, Feng Gao, Monica Lira‐Cantu

**Affiliations:** ^1^ Catalan Institute of Nanoscience and Nanotechnology (ICN2) CSIC and BIST Barcelona Catalonia Spain; ^2^ Department of Physics and Astronomy ‘Ettore Majorana' University of Catania, and CNR‐IMM Catania Italy; ^3^ Departament de Fisica, Facultat de Ciencies Universitat Autonoma de Barcelona (UAB) Barcelona Spain; ^4^ ICREA Barcelona Catalonia Spain; ^5^ Department of Physics Chemistry and Biology (IFM) Linköping University Linköping Sweden

**Keywords:** interface engineering, inverted perovskite solar cells, MXene, stability, thermal conductivity

## Abstract

Despite inverted perovskite solar cells (iPSCs) having reached power conversion efficiencies (PCEs) of about 27% with long‐term stability at room temperature, their commercial devices must prevent heat‐induced performance degradation. In PSCs, 2D MXenes have been employed to enhance their long‐term stability at high temperatures; however, a deep understanding of the heat management mechanism of applying MXenes at interfaces is still lacking. Herein, we investigate the application of MeO‐2PACz‐functionalized MXenes at the hole transport layer (HTL) interface in iPSCs using MeO‐2PACz as the HTL. PSCs reached PCEs of 23.02 ± 1.14%, observing a 400% enhancement in their operational stability, spanning the T_80_ lifetime at 65°C from 141 to 687 h. HAADF‐STEM imaging and STEM‐EELS analysis confirmed the effective intercalation of the MeO‐2PACz within the MXene atomic nanolayers. Thermal conductivity measurements and simulations revealed that the application of the functionalized MXenes can effectively dissipate the heat from the perovskite absorber, reducing the perovskite temperature by about 20 ± 6%. Overall, the fourfold extension of T_80_ by using MeO‐2PACz‐functionalized MXenes in iPSCs is attributed to the synergy between reduced thermal accumulation and improved buried‐interface quality, including defect passivation, enhanced charge extraction, and suppressed ion migration.

## Graphical abstract

MeO‐2PACz‐functionalized MXene nanosheets create a thermally conductive buried interface in inverted perovskite solar cells. The interface improves heat spreading, suppresses defects and ion migration, and strengthens charge extraction, extending T80 at 65°C from 141 to 687 h while maintaining competitive efficiency. The study reveals how thermal and interfacial engineering act together to stabilize perovskite photovoltaics.

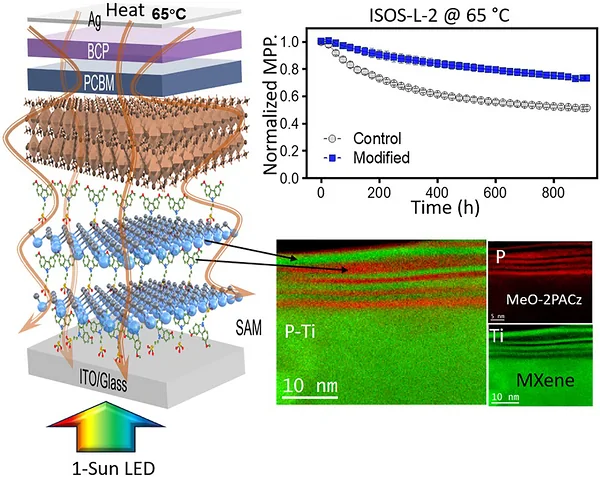

## 1 Introduction

Perovskite solar cells (PSCs) have gained significant attention due to their exceptional power conversion efficiencies (PCEs) and relatively low‐cost fabrication processes [1]. Among the various architectures, inverted perovskite solar cells (iPSCs) are strong candidates due to the possibility of being applied in tandem PSC configurations, taking advantage of their improved low‐temperature performance and ease of fabrication [2], surpassing normal‐configuration PCEs above 26% [3, 4, 5], stability, and compatibility with flexible substrates [6]. However, a critical area of research is still their long‐term stability, particularly at high temperatures (above 55°C) [1, 7, 8]. Thermal management in PSCs is highly important not only to surpass the ISOS protocols [9] and IEC standards (especially IEC 61215 [10]) required for their commercialization, but also for the potential of PSCs to be employed in novel applications in extreme environments like space, building integration, or reverse solar cells [11].

Several strategies have been employed to improve the efficiency and stability of PSCs; among them are the surface and interface passivation of defects employing molecular [3, 12], polymeric [13], and 2D materials [14, 15]. These methods aim to suppress trap states and reduce interfacial defects, contributing to notable improvements in PCE and stability [2]. Passivation through self‐assembled monolayers (SAMs) has recently been the focus of attention [16, 17, 18, 19] for the modification of the energy alignment and reduction of surface defects [2, 20, 21, 22, 23]. By tuning the molecular structure of SAMs, it is possible to optimize the alignment of the energy levels between the perovskite absorber and the adjacent transport layers [2]. As a result, iPSCs fabricated with SAMs have demonstrated enhanced long‐term stability, retaining a significant portion of their initial efficiency even after extended periods of operation under real‐world conditions [24, 25, 26, 27]. The use of organic cation‐based passivating agents has been reported to stabilize the perovskite crystal structure at elevated temperatures [28]. For example, the incorporation of ionic liquids [29] and cross‐linkable passivation layers [5, 13] has been shown to improve resistance to thermal decomposition by reinforcing the lattice structure and enhancing tolerance to temperature variations. Similarly, the use of fluorine‐containing passivation materials has been shown to enhance thermal stability by creating hydrophobic layers that prevent moisture ingress and reduce the likelihood of ion migration under high‐temperature conditions [30, 31, 32, 33]. Another strategy recently employed is the application of two‐dimensional (2D) materials (e.g., Graphene, MXenes, MoS_2_, h‐BN) that have received significant attention due to their high thermal conductivity and thermoelectric properties [34], granting excellent thermal energy conversion and management [34]. Among them, MXenes have shown promising thermal property values. Chen et al. [35]. estimated a thermal conductivity of 2.84 W/(m∙K) at 290 K in Ti_3_C_2_T_X_ films using a state‐of‐the‐art T type method. Another study by Liu et al. [36] reported an enhanced thermal conductivity in Ti_3_C_2_T_x_/PVA composites of up to 55.8 W/(m∙K) using Raman thermometry. Wang et al. [37] predicted high thermal conductivity values up to 140 W/(m∙K) for Ti_3_C_2_T_x_ of different compositions using a molecular dynamics model [37].

In PSCs, the use of MXenes has been employed to improve optoelectronic properties and long‐term stability [14, 33, 38, 39, 40]. However, only a few works analyze the stability of PSCs under continuous light irradiation at high‐temperature conditions [33, 40, 41]. For example, Heo et al. mixed oxidized MXene into the inorganic CsPbI_3_ perovskite film composition in an inverted configuration with good thermal stability over 1000 h using ISOS‐T‐3 protocol [41]. They demonstrated good thermal stability (ISOS‐T), with the PSCs surpassing the standard IEC 61215, unfortunately, the authors did not perform lifetime stability studies under continuous light irradiation at high temperature (ISOS‐L). They attributed the improved performance of solar cells to the effective charge transfer between TiO_2_ anatase formed on oxidized Ti_3_C_2_ MXene surfaces and perovskite grains. Yakusheva et al. used MXene:Bathocuproine (BCP) as ETL in iPSC with ITO/NiO_x_/CsFAPbI_3_/C_60_/MXene:BCP/Ag configuration. They demonstrated that the BCP:MXene layer enhanced the efficiency of the electron collector, as well as the conductivity, stabilizing shunt properties and rectification due to the passivation of shunt defects. As a result, highly stable PSCs with PCE of 17.5% and working at 63°C (ISOS‐L) extended the operational stability of devices from T_80_ = 400 h to > 2300 h when applying MXenes under the ISOS‐L‐2 protocol [33]. These works attributed the stability enhancement of the PSCs to the defect passivation properties and high conductivity of MXenes [41, 42]. The only example where the thermal management properties of MXenes [40] were considered, the PSCs were analyzed at high temperatures (ISOS‐T) without the use of constant light irradiation (ISOS‐L). In this case, Wan et al., analyzed normal configuration PSCs with MXenes being employed together with the halide perovskite, both in the absorber layer. The authors emphasized the multifunctional effect of MXenes, especially the improvement observed due to the thermal conductivity of MXenes. They reported T_80_ of about 500 h using the ISOS‐T protocol at 85°C [40].

In this work, we want to highlight the thermal dissipating effect of 2D MXenes when employed as HTL in a PSC, and its impact on the long‐term stability of PSCs analyzed under continuous light irradiation at 65°C (ISOS‐L). The devices were also analyzed under outdoor conditions (ISOS‐O). For this, we carried out the intercalation of the HTL molecule, (2‐(3,6‐Dimethoxy‐9H‐carbazol‐9‐yl)ethyl)phosphonic acid (MeO‐2PACz), within the Ti_3_C_2_ MXene interatomic layers (nanosheets of 5–7 nm thickness). The functionalized MXene:MeO‐2PACz nanosheets were employed as an interface between the 3‐cation perovskite absorber layer and the ITO/HTL substrate. The devices modified using MXene:MeO‐2PACz have a device configuration of ITO/HTL/MXene:MeO‐2PACz/3‐cation perovskite/BCP/PCBM/Ag. Champion solar cells obtained a PCE of 24.16% and showed a 4‐fold enhancement in high‐temperature operational stability (ISOS‐L‐2@ 65°C) with T_80_∼687 h. Thermal conductivity analyses carried out on the PSCs demonstrated that the MXene:MeO‐2PACz interface is able to reduce the heating effects, acting as heat dissipators and improving the thermal stability of the solar cells. In addition, the enhanced optoelectronic properties of the MXene‐modified PSCs are attributed to the improved crystallinity, band alignment, improved charge injection, and reduced deep and shallow trap states.

To our knowledge, this work is the first to report the thermal dissipation properties of MXenes applied in inverted PSCs as the HTL and analyzed under continuous light irradiation conditions at high temperature (ISOS‐L‐2) and under outdoor conditions (ISOS‐O). We hope that our work can shed light on the heat dissipation mechanisms that characterize two‐dimensional (2D) materials (e.g., 2D halide perovskites, graphene), which have demonstrated good thermal stability at 85°C but whose underlying thermal dissipation mechanisms have not yet been fully explored in PSCs.

## 2 Results and Discussion

### 2.1 MXene Nanosheet Functionalization and their Deposition on ITO/HTL Substrates

**FIGURE 1:**
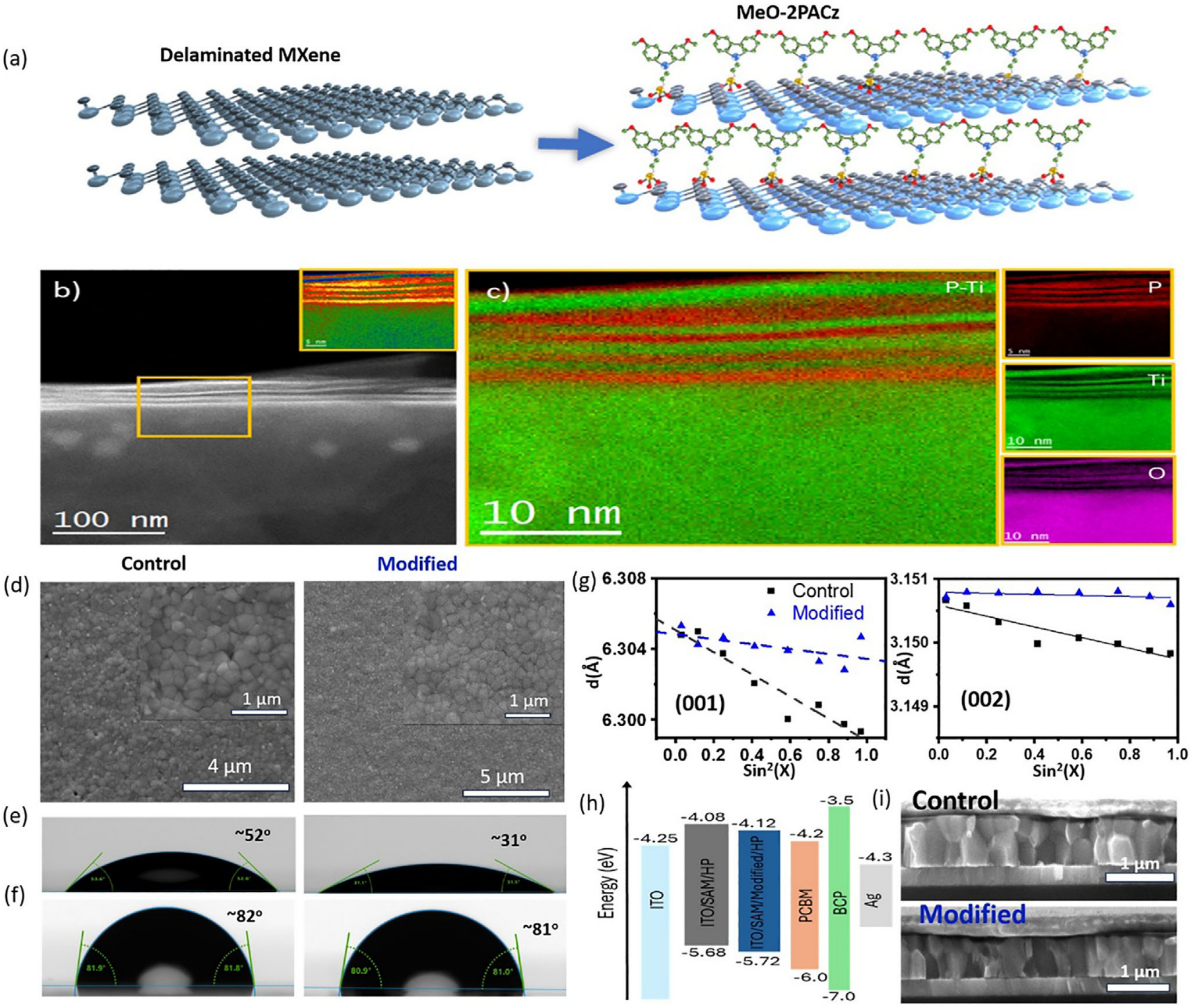
(a) Schematic of cross‐section interlayer insertion of MeO‐2PACz molecules into MXene nanosheets, (b) HAADF‐STEM image of the layered Ti_3_C_2_ ultrathin nanoflake. In the inset, a blow‐up image of the region in the yellow box showing the P (representative of MeO‐2PACs molecule) signal in temperature color palette. (c) STEM‐EELS color map showing the p and Ti signals together, in red and green, respectively, and individual signals including O in pink. The ionization energy edges considered for EELS analyzes are: Ti L‐edge at 456 eV, O K‐edge at 532 eV, and P K‐edge at 214 eV, (d) Comparison of surface SEM images of the perovskite grains (inset: SEM images in 1 µm scale) deposited on Modified and Control ITO/HTL substrates (e) Contact angle measurements of ITO/HTL (left) and ITO/HTL/MXene: MeO‐2PACz (right) substrates, (f) Contact angle measurements of perovskite films deposited on ITO/HTL/perovskite (left) and ITO/HTL/MXene: MeO‐2PACz/perovskite (right) substrates, (g) Comparison of XRD χ‐scan of perovskite Bragg peaks of (001) and (002) for Control and Modified films, (h) Calculated energy band diagram for Control (ITO/HTL/HP) and Modified (ITO/HTL/MXene: MeO‐2PACz/HP) devices, (i) Cross‐section SEM images of the devices.

Figure 1a shows the schematic of insertion of MeO‐2PACz molecules into the interlayers of delaminated MXene nanosheets. Figure 1b,c shows a thin nanosheet of Ti_3_C_2_ (with 4 atomic layers: 6–8 nm thickness) that is atomically functionalized with the MeO‐2PACz molecule in correspondence with the designed schematic of Figure 1a. The scanning transmission electron microscopy image acquired in high angle annular dark field mode (STEM‐HAADF) shows intra‐layer planes of around 1.4‐2Å. As it can be clearly observed in the electron energy loss spectroscopy map acquired in STEM (Figure 1c), the Phosphorous (P) atomic distribution takes place exactly in between the Titanium (Ti) interatomic layers, which indicate the direct observation of the intercalation of the MeO‐2PACz molecules within the Ti_3_C_2_ MXene atomic layers. The signal coming from the P is complementary to the one coming from Ti, confirming the inter‐layer location of P. The oxygen signal also appears more intense in the inter‐layer regions (Figure 1b,c). After MXene delamination, the effective functionalization was analyzed, as shown in Figure S1. It is crucial to obtain a very thin and stable solution of the MXene nanoflakes for a uniform functionalization of their surface with MeO‐2PACz. For this, we have employed TPAOH [14] and LiCl as both scissoring and etching agents simultaneously. Figure S2a,b shows the XRD of bulk, delaminated, and repeated functionalization of MXene nanoflakes and TEM of delaminated MXene nanosheets, respectively [38].

After the intercalation of the MeO‐2PACz molecule within the MXene nanosheets, we analyzed the interface passivation between HTL layer and the perovskite film. In this report, the HTL corresponds to the spin‐coated MeO‐2PACz employed as HTL (with an optimized value of 3 mg/mL in Isopropyl alcohol, IPA, solvent) on pre‐cleaned ITO substrates (See Supporting information for experimental details). Thereafter, the prepared solution of MXene:MeO‐2PACz nanoflakes was deposited on top of the HTL layer. Figures S3 and S4 show the surface SEM images of the functionalized MXene deposited on top of the ITO/HTL substrates. As it can be observed, the MXenes nanosheets are flat and are extensively extended in an in‐plane geometry and thus are well attached to the ITO surface. Figure S4a,b shows an example of a large (scale of 40 µm) and flat MXene flake with the corresponding energy‐dispersive spectrum and mapping images to verify it as MXene Ti_3_C_2_ with homogenous distribution of P and O atoms, in agreement with the MeO‐2PACz molecule composition. This deposition is very important to be smooth and flat, because any roughness induced by MXenes can influence the perovskite layer deposition and the resultant roughness, that is not favorable for good device performance. To ensure the correct roughness and topography of these nanosheets, we analyzed them by atomic force microscopy (AFM), as shown in Figure S5. The AFM images in Figure S5a show the effective attachment of the MXene:MeO‐2PACz nanosheets to the surface of ITO, and the average roughness across the nanosheet, respectively. The average roughness of ITO modified with MXene:MeO‐2PACz is estimated to be about 10–20 nm, as indicated by the 3D image in Figure S5b, which indicates that the modified substrate of ITO/SAM does not have a rough surface.

We further provided surface SEM images of perovskite films deposited on the Control (non‐modified ITO/HTL) and modified ITO/HTL substrates in Figure 1d. It is observed that the control device has ordered grains on a small scale with pinholes (inset Figure 1d), but in larger views up to 4 µm scale, more pinholes are observed. On the other hand, the perovskite film on the modified substrates shows highly ordered grains and is pinhole‐free at large scales of 5 µm. Interestingly, the contact angle measurements for ITO/HTL substrate and ITO/HTL/ MXene:MeO‐2PACz are compared in Figure 1e, respectively. It is inferred that this MXene:MeO‐2PACz modification results significantly in the reduction of the contact angle from 53 ± 0.5° to 31 ± 0.6° and the increase of wettability of surface which is crucial for well distribution of perovskite solution, nucleation and crystal growth to obtain pinhole free films [44]. Moreover, contact angle measurements of perovskite films for Control and Modified MXene:MeO‐2PACz on ITO/HTL substrates are shown in Figure 1f, which indicate values of 81.9 ± 0.1° and 80.8 ± 0.1°, respectively. This suggests that, in addition to the fact that MXene:MeO‐2PACz on ITO/HTL increases the wettability of the substrate for better perovskite film deposition and resulted in higher quality pinhole‐free perovskite film, it also preserves roughly the same hydrophobic properties on the surface of the perovskite film.

To pursue this, XRD plots of the films were provided in Figure S6a. It shows that the modified perovskite film has less PbI_2_ phase intensity (Figure S6a inset) and a higher perovskite preferential growth peak of (001) compared to the Control film, with decreased growth peak for (011) and (220) atomic planes, as indicated by the arrows. This change of crystal growth towards (001), together with the decrease of the PbI_2_ phase, can result in better film growth with improved crystallinity and reduced lattice strain [45, 46, 47]. To study in depth, a series of x‐ray diffraction Chi‐scan were performed for the selected Bragg peaks of (001), (002), and (220) in the range of χ = 0–80° [12, 47] as shown as example in Figure S6b. Figure 1g and Figure S6c show the resulting strain plots with the corresponding linear fit for (001), (002), and (220) Bragg peaks, respectively. It can be clearly seen with the interfacial modification, the compressing strain (negative slope) for (001) and (002) reduces significantly, whereas for the (220) it induces a small stretching strain (with almost positive slope). This could be due to better lattice matching between MXene as a 2D material in out‐of‐plane crystal growth geometry and perovskite with out of plane favoring growth crystallites. Thus, it can refer to the peak intensity variations for these peaks. In addition to that, Williamson–Hall analysis [48] was also performed to reveal any reduced nonuniform lattice strain due to this modification shown in Figure S6d. It shows a reduction of lattice microstrain from 0.75 × 10^−3^ to 0.64 × 10^−3^. The reduction of nonuniform strain is very crucial in the suppression of void formation and pinholes in the growth of grains [49].

To scrutinize more on the electronic effect of MXene:MeO‐2PACz deposition on ITO/HTL substrates, ultraviolet photoelectron spectroscopy (UPS) measurements were performed to determine the Fermi level and valence band position of bare ITO, ITO/HTL (MeO‐2PACz), ITO/HTL with bare MXene deposited on top of it (for comparison purposes), and ITO/HTL modified with MXene:MeO‐2PACz nanosheets in Figure S7a,b, respectively, and the calculated values are summed up in Table S1. It shows clearly that due to MXene:MeO‐2PACz nanosheet modification, the Fermi level shifts slightly downward, meaning to favor *p*‐type properties of the resultant film, and the valence band position also shifts significantly from −5.29 eV for ITO/HTL to −5.53 eV for ITO/HTL/MXene:MeO‐2PACz film. This shift indicates that the charge injection enhancement is plausible from the valence band of perovskite to the valence band of ITO/HTL layer. To further confirm it, UPS analyzes were also conducted for the 3‐cation perovskite film on non‐modified and Modified substrates (Figure S7c,d), and the resultant band diagram is plotted in Figure 1h. As it is shown, the Modified sample valence band is shifted to lower energy, which means that the hole injection to ITO fermi level can be facilitated, considering the fact that the valence band edge of perovskite is shifted towards the valence band of ITO/HTL/Modified perovskite film, and also the electron injection is enhanced due to shifting of CB from −4.08 to −4.12 eV which is closer to −4.2 eV of PCBM layer therefore the charge injection improvement is favorable. Figure 1i shows the cross‐SEM images of selected Control and Modified devices to stress the similarity of layering order, roughness, and perovskite film growth for both types of devices indicating that this modification does not induce any roughness or non‐homogeneity in the growth of perovskite layer and, consequently, the device configuration.

### 2.2 Device Performance and Stability Assessment

As described above and in the Supporting Information, the MXene:MeO‐2PACz was dispersed in IPA, we also employed IPA as the solvent for the deposition of the SAMs molecule as HTL, ensuring solvent compatibility. Therefore, a series of devices were fabricated using different concentrations of MeO‐2PACz as the SAM layer (3 and 5 mg/mL), and the results are plotted in Figure S8. The best concentration was 3 mg/mL from both efficiency and ISOS‐D‐1 stability progression. In this work, we refer as “Control” to the PSCs fabricated using a concentration of 3 mg/ml of MeO‐2PACz as HTL layer on ITO substrates. All the other devices employing different concentrations are labelled “Modified”.

**FIGURE 2:**
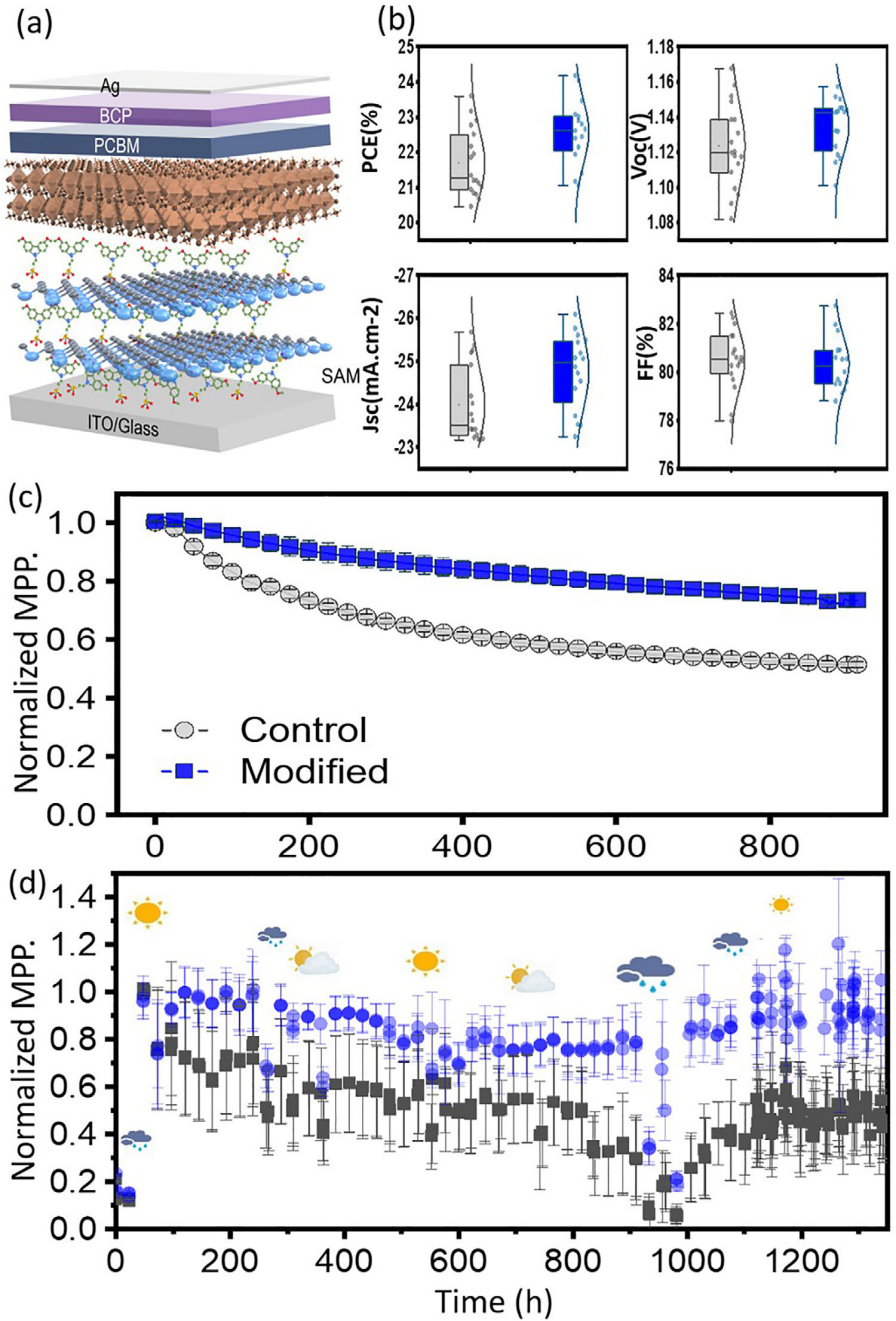
(a) Schematic of the Modified device configuration in inverted structure, (b) Devices’ parameter comparisons among 24 devices per group, (c) Stability assessment of un‐encapsulated devices (12 devices per group) with averaged values for each group using ISOS‐L‐2 protocol under continuous simulated 1‐Sun illumination, at constant 65°C, under N_2_ atmosphere for 1000 h, (d) Outdoor averaged stability of encapsulated devices (ISOS‐O‐2) by means of MPP tracking for 6 devices per each group conducted at ICN2, UAB, Barcelona, Spain during February–April 2025.

Figure 2a shows a schematic representation of the inverted perovskite solar cell based on the MXene:MeO‐2PACz employed as an interfacial passivator. Figure 2b shows the comparison of the statistical distribution of the device performance parameters across different batches for the modified and non‐modified devices. The results show a slight improvement in the performance of the modified device compared to the Control sample; we attributed this response mostly to the improvement observed in fill factor (FF) and short‐circuit current density (Jsc), and the minor improvement observed in open‐circuit voltage (Voc). The devices were set for a stability analyses as shown schematically in Figure S9a, by applying the ISOS‐L‐2 protocol, where samples were analyzed at the maximum power point (MPP) every 6 min under continuous light soaking under the simulated 1‐Sun LED lamp, at a constant temperature of 65°C, under an inert N_2_ gas flow atmosphere (unencapsulated samples) for 1000 h. Figure 2c shows the average of the MPP normalized values for each category (12 devices per category). The results show that devices in each category have excellent consistency in stability, with roughly constant minimal error bars across the whole time of measurement, which indicates good reproducibility. Moreover, the modified device shows excellent operational stability enhancement under an elevated temperature of 65°C, with T_80_ of about 687 h, while the Control devices show a maximum of 141 h [40, 50].

To further analyze the stability of the PSCs when the MXene is employed, we carried out stability analysis under ISOS‐O protocol (outdoor analysis). Thus, the samples (6 devices per category) were first encapsulated as explained in the experimental section and placed on the rooftop of the ICN2 building, Barcelona, Spain. Figure 2d shows the averaged normalized MPP values of the devices extracted during the first 1400 h, with recorded daily temperature, humidity, and irradiance for each device (Figure S9b). The modified samples show substantial stability, retaining about 96%–98% of their initial PCE after 1400 h, while the Control devices retain less than 50% of their initial MPP after the same testing period. These remarkable results indicate the effectiveness of simple interface passivation using the MXene‐MeO‐2PACz nanosheets.

The stability at room temperature of PSCs employing MXenes has already been reported in the literature [38, 40]. In this work, however, we have demonstrated enhanced stability under more aggressive testing conditions, which is under continuous light irradiation at a temperature of 65°C. To the best of our knowledge, this is the first report where functionalized MXenes are employed at the interface of the HTL and the Halide perovskite and assessed for high‐temperature stability (See Table S2). To enable a meaningful comparison, Table S2 distinguishes the MXene position and the applied ISOS protocol. Reported MXene‐based PSCs span markedly different test conditions, including dark thermal aging [40], continuous illumination at room temperature, and outdoor operation. For example, recently Wei et al. have fabricated IPSCs with Annealed Ti_3_C_2_ MXenes (A‐Ti_3_C_2_) between ITO and SAM (4‐PhCz) layers to enhance the performance of the devices. Their best stability record is ISOS‐T‐1 for 500 h, where the device target retains 83.68% of the initial PCE [50]. Wan et al. also reported thermal management using MXenes mixed with a perovskite film, with improved stability spanning T80 of 500 h and 350 h using ISOS‐T‐1 and ISOS‐L‐1 protocols, respectively [40]. The present study provides T_80_ = 687 h at 65°C under ISOS‐L‐2 assessment and retains approximately 95%–98% of its initial performance after 1400 h of encapsulated ISOS‐O‐2 tracking. In addition, Table S3 provides a summary of ISOS‐L‐2 protocol to scavenge between different strategies for the enhancement of Inverted perovskite solar cell stability. Some reports indicate the stability span of the devices over 2000 h for IPSC when using NiO_x_ as HTL [6, 33, 51, 52]. Some reports with solely using SAM molecules have ISOS‐L‐2 stability reports of about 2000 h [53]. For example, Huang et al. have found that positional methylation isomers can actually anchor to the perovskite and regulate active sites, leading to high efficiency and stability of IPSCs [53]. Therefore, in this scenario, SAM‐based IPSCs and functionalized MXenes could be a possible solution for buried interface passivation and prolonging the stability of solar cells under multiple degradation stressors by further manipulating functional molecules, type of MXenes, and their morphological adjustments.

### 2.3 Electrical Characterization of the Devices

**FIGURE 3:**
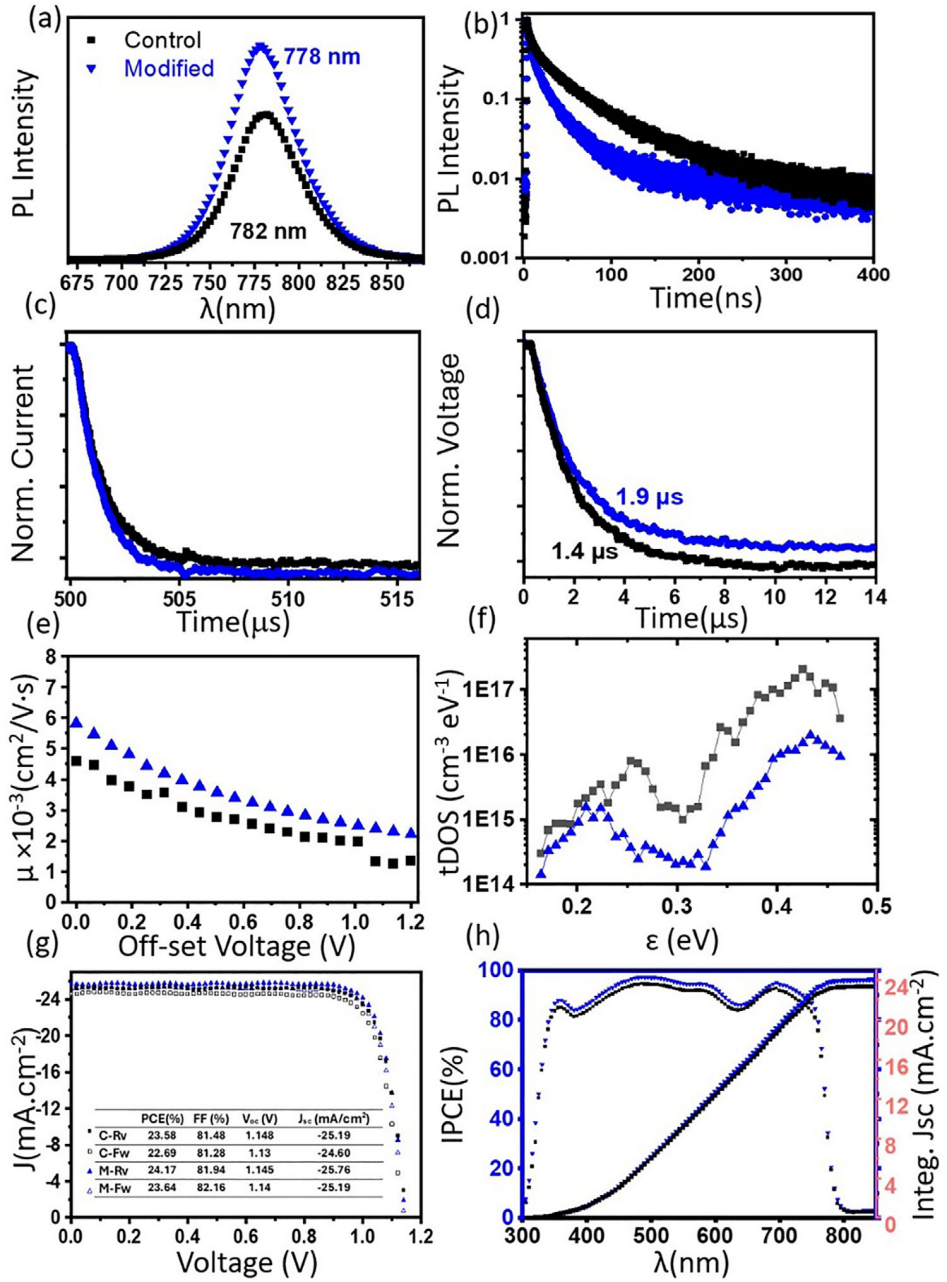
(a) Photoluminescence (PL) of perovskite deposited on Glass/HTL (Control) and Glass/HTL/ MXene:MeO‐2PACz substrates, and (b) Time‐resolved Photoluminescence (TRPL) of perovskite deposited on ITO/HTL (Control) and ITO/HTL/MXene:MeO‐2PACz substrates, (c) Transient photocurrent (TPC), and (d) Transient photovoltage (TPV) spectroscopy of the devices at 10% and 100% of simulated 1‐Sun intensity, respectively, (e) Calculated mobility (µ) of devices for different offset applied voltages, (f) Calculated Trap density (tDOS) vs. conduction band position as zero using Thermal admittance spectroscopy, (g) Reverse (Rv)‐Forward(Fw) scans of J‐V curves of champion Control (C‐Rv, C‐Fw) and champion modified device (M‐Rv, M‐Fw), respectively, and (h) IPCE (left axis) and calculated integrated Jsc (right axis) plots of the selected champion devices.

Figure 3a shows the photoluminescence (PL) spectra of the perovskite films deposited on glass/HTL with (Modified) and without (Control) the use of the HTL molecule MXene‐MeO2PACz. Our results show an increase in the PL intensity for the Modified sample, with a small blueshift in the PL emission peak, from 782 to 778 nm. As inferred from XRD analyses, this blueshift can be corelated to the structural reorientation growth of the halide perovskite [54], and consequently a slight increase in the band gap value of the perovskite [55]. Therefore, these observations also refer to the reduction of sub‐band trap states and band tails that leads to the blueshift of the PL spectrum, in agreement with observations from Westbrook et al. [56] Moreover, the increase in the intensity of the PL could be attributed to the passivation of buried interface defects [12]. Figure 3b shows the time‐resolved PL (TRPL) of perovskite film for the Modified and the Control (nonmodified) samples on ITO/HTL substrates. In this case, we used ITO/HTL to explore the effect of passivation in terms of enhancing charge extraction and therefore increasing the rate of the PL signal decay. This indicates that the faster decay observed in the PL intensity over time is either due to non‐radiative recombination or to charge separation/transfer to the ITO layer. Since the PL proved the passivation of non‐radiative recombination, the only possible mechanism is the faster charge transfer rate for the modified sample. To further investigate this idea, transient photocurrent (TPC) spectroscopy was performed for the devices. Samples were measured under 10% of 1‐Sun intensity as shown in Figure 3c. Results show an enhanced charge extraction rate and faster decay for the Modified device [57]. Figure 3d also shows the transient photovoltage decay of the selected devices, which indicates that the modified device preserves the induced voltage across the device for longer periods of time, which is attributed to less recombination rates of carriers [58]. These results indicate faster charge transfer and reduced trap density states for the Modified device. To understand how these results affect carrier mobility, we carried out charge extraction by linearly increasing voltage (CELIV) analyses measured across the different offset voltages. The results, shown in Figure 3e, show an enhancement in the mobility values of the Modified device across all ranges of applied voltages. This enhancement is more pronounced at low voltages, close to the shunt regime, and it has a slight decrease at the high voltages that are close to the open‐circuit values. This is a clear indication of trap densities reduction at the interfaces that boost charge extraction on the HTL layer. To confirm the reduction in trap density, thermal admittance spectroscopy (TAS) analysis was carried out to the samples. Figure 3f shows that the defect density on the buried interface has been reduced to less than 10% in the range of 0.22 to 0.45 meV below the conduction band. These results are an indication of significant passivation of trap states that are responsible mainly for charge extraction enhancement and significant operational stability improvements [26]. JV plots of two champion PSCs cells are presented with reverse/forward measurements, and their corresponding cell parameters are shown in Figure 3g. We observed that the modification using MXene:MeO‐2PACz allows for a slight decrease of the Hysteresis index (HI) from 3.8% for the Control samples to 2.1% for the Modified device. It is worthy to mention that this HI value is comparable to recent MXene/perovskite interface study by Wei et al. [50] and even lower than reported values for HTL based reports by Zhao et al. 2.6%–5% [59], and for Randi Azmi et al. 6.8% [60]. The JV curves indicate that this decrease in the HI value is due to enhancement of the current density of reverse‐forward measurement for the modified device. Moreover, FF values for both reverse and forward scans are about 82% for the active area of 0.16 cm^2^, which are quite high for HTL based inverted perovskite solar cells [59, 60]. To emphasize on the enhancement of charge extraction, the incident photon‐to‐current efficiency (IPCE) of both devices is shown in Figure 3h, which indicates a slight enhancement of the integrated current density values for the Modified device. Calculated Integrated J_SC_ values are 24.07, and 23.35 mA.cm^−2^ for Modified and Control devices, respectively. The corresponding values from the JV curves are 25.76 mA.cm^−2^ from the reverse scan (25.15 mA.cm^−2^ from the forward scan) and 25.19 mA.cm^−2^ from the reverse scan (24.60 mA.cm^−2^ from the forward scan). Expressed as the ratio *J*_SC, JV_/*J*_SC, IPCE_, the discrepancy values are approximately 1.070 for the reverse scan and 1.045 for the forward scan, corresponding to relative differences of approximately 7.0% and 4.5%, respectively. When the reverse and forward scans are averaged, the JV‐derived current is approximately 5.8% higher than the integrated IPCE current. The remaining 4.5%–7.0% difference is consistent with the systematic tendency of JV‐derived *J*_SC_values to exceed integrated EQE values in perovskite solar cells studied by Saliba et al. [61]. This difference may arise from the distinct spectral, illumination‐intensity, steady‐state, and voltage‐sweep conditions employed in the two measurements. Importantly, a similar discrepancy is observed for both the Control and Modified devices, and therefore it does not affect the comparative enhancement produced by the MXene:MeO‐2PACz interface. Overall the electrical characterization results indicate that this modification has a crucial effect on both deep and shallow trap state passivation, faster charge extraction, and therefore enhanced carrier mobility, with a noticeable enhancement in HI reduction due to increased charge collection. We have performed post‐stability assessment analyzes for the champion devices shown in Figures S10–S12 with a relevant discussion in Section S2.4 to further emphasize the role of this interface engineering on device operational stability. Cross‐sectional SEM after 1000 h of ISOS‐L‐2 testing shows that the modified device preserves a compact and attached buried interface, whereas the Control device exhibits localized degradation and delamination. Post‐stability impedance analysis further indicates suppressed ionic accumulation at the Modified interface. These observations support the operational integrity of the functionalized MXene interface.

### 2.4 Thermal Transport Assessment of ITO/Perovskite/MXene:MeO‐2PACz System

**FIGURE 4:**
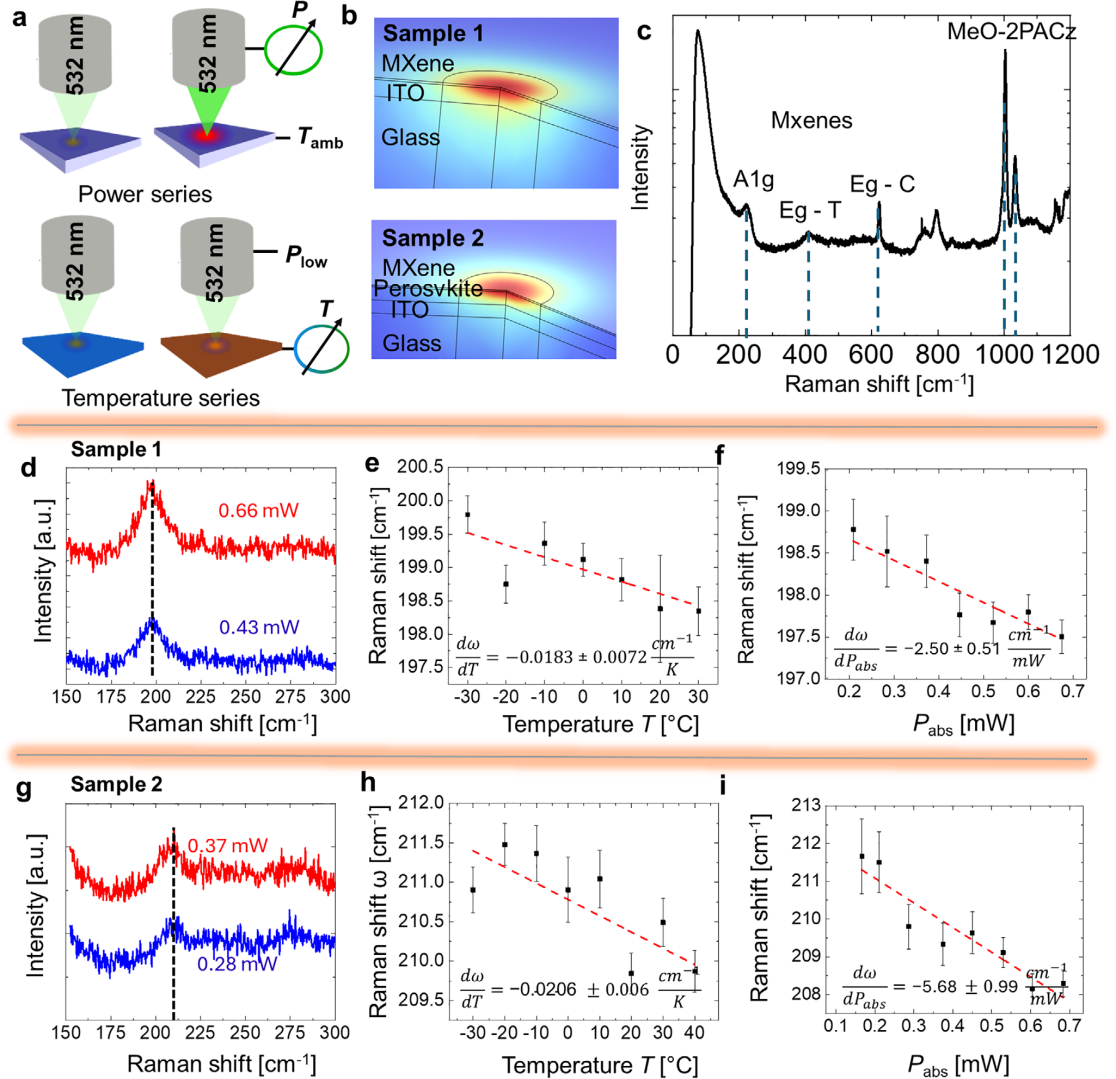
(a) Schematic of the power and temperature series measurements, where the Raman shift of the sample is investigated first as a function of the applied laser power at ambient temperature and second by varying the stage temperature while keeping the laser power constant, (b) Temperature distribution schematic of the two samples under investigation around the laser spot, showing the structure without and with the perovskite layer, (c) Raman spectrum of the studied MXene obtained over a broad Raman shift range. The characteristic MXene peaks at approximately 205, 408, and 616 cm^−1^ along with those corresponding to MeO‐2PACZ, are highlighted, (d–f) Temperature measurements on Sample 1: (d) Confined Raman spectra showing the characteristic MXene peak around 205 cm^−1^ at two different power magnitudes resulting in a red shift at higher power, (e) Graphical illustration of the MXene peak position as a function of the stage temperature *T*, (f) Graphical illustration of the MXene peak position as a function of the absorbed laser power *P*_abs_, (g–i) Temperature measurements on Sample 2: (g) Confined Raman spectra showing the characteristic MXene peak around 205 cm^−^^1^, (h) Graphical illustration of the MXene peak position as a function of the stage temperature *T*, (i) Graphical illustration of the MXene peak position as a function of the absorbed laser power *P*_abs_.

Figure 4a,b illustrates schematically the Raman thermometry (LRT) setup employed for the thermal characterization [62]. With this approach, we examined the two systems of ITO/perovskite/ MXene:MeO‐2PACz and ITO/ MXene:MeO‐2PACz in terms of heat spreading of the MXene layer. This was done by analysing the temperature‐dependent red shift of the Raman ω peak of the Raman spectrum, in two ways. First, we heat the samples by increasing the power magnitude of the laser probe (532 nm). Second, we heat and cool the sample externally using a temperature‐controlled sample stage (Linkam). By that means, we can extract the temperature increase of the MXene layers as a response to the applied power *P*_in_ using Equation (1).

(1) $$\Delta T=\frac{\omega /P_{in}}{\omega /T}\;\;\left[\frac{K}{mW}\right]$$

In the first set of measurements, we examined ITO/MXene:MeO‐2PACz samples without the halide perovskite layer. Figure 4c presents the Raman spectrum of this sample obtained at a large range from 50 to 1200 cm^−1^. From this spectrum, we can extract different peaks related to the material under investigation. We identified three peaks associated with the MXene at approximately 205, 408, and 616 cm^−1^. These peaks correspond to the out‐of‐plane A_1g_ vibrational mode from carbon atoms, the Ti layers, and the functional groups (205 cm^−1^), from E_g_ in plane vibration of the functional groups (408 cm^−1^), and from E_g_ in plane vibration of the carbon atoms (616 cm^−1^) [64, 65].

In the subsequent measurements, we focused on the A_1g_ peak at 205 cm^−1^, as it is the most prominent across the samples. Analysing the ITO/MXene:MeO‐2PACz nanoflakes sample, the MXene peaks exhibit minor to medium power dependencies. Figure 4d shows the Raman spectra in one of the nanoflake samples for two different power magnitudes. Figure 4e,f shows the fitted peak position as a function of the temperature and the absorbed laser power *P*_abs,_ respectively. In this instance, a clear correlation between the Raman shifts and the absorbed laser power is visible, showing the maximum slope observed for this sample (−2.5 ± 0.51 cm^−^^1^/mW). Following Equation 1 this would result in a temperature increase of around 137 ± 28 K/mW. In this sample, the laser light is predominantly absorbed by the MXene as the substrate is transparent.

In the ITO/perovskite/ MXene:MeO‐2PACz sample, where the perovskite layer is sandwiched between the ITO and the MXene, the power dependency of the Raman shift is more pronounced. Each nanoflake exhibited a clear power dependency with an average slope of −4.34 ± 1.93 cm^−^^1^/mW. Figure 4g–i shows the results of the Raman spectra in a MXene nanosheet of the ITO/perovskite/MXene:MeO‐2PACz sample. The higher power slope signifies an enlarged temperature increase in the MXene of around 219 ± 98 K/mW. In this instance, additional heating is produced by the absorbed laser power in the perovskite.

The temperature series of both samples, as illustrated in Figure 4e,h, shows a continuous red shift and displays comparable temperature calibration values. This matches our expectations, as the temperature series should intrinsically depend on the MXene material and thus is not affected by the perovskite. Additionally, it shows that the red shift of the Raman peak can be attributed to a variation in temperature. From this observation, we can conclude that excess heat generated from the perovskite layer results in an increased temperature of the MXene region. The temperature rise in the MXene layer hereby depends on the thermal conductivity of the MXene.

**FIGURE 5:**
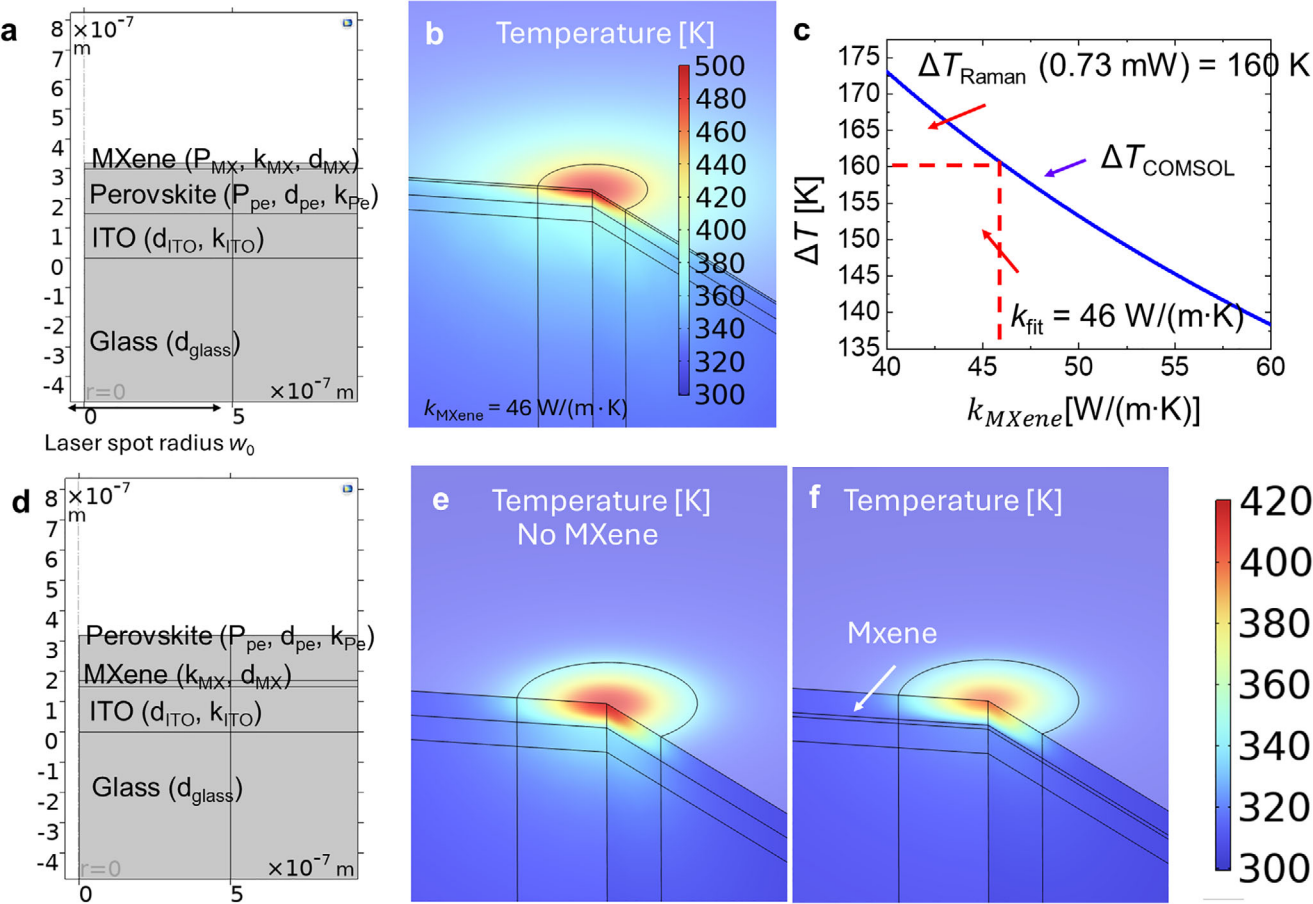
(a–c) COMSOL model for the assessment of the thermal conductivity of the MXene layer in Sample 2. (a) Geometry of the investigated layer structure, (b) 3D Temperature distribution around the laser spot radius for a thermal conductivity of the MXene of *k*_MXene_ = 46 W/(m∙K). (c) Calculated temperature increase of the MXene Δ*T* at the laser spot area at an absorbed power of 0.73 mW as a function of the model parameter *k*_MXene_. The results are fitted with the experimental Raman data to extract the thermal conductivity of the MXene, (d–f) COMSOL model to assess enhanced heat spreading in a device cell structure, (d) Geometry of the device structure, (e,f) 3D Temperature distribution around the laser spot radius for a thermal conductivity of the MXene of *k*_MXene_ = 46 W/(m∙K) at an arbitrary power in the structures (e) without MXene and (f) with MXene.

To assess the heat‐dissipating potential of MXene, we employ a COMSOL finite element method (FEM) model to estimate the thermal conductivity of the MXene layer. All the simulation parameters can be found in Table S4. For this model, we replicate the Glass/ITO/Perovskite/MXene structure in accordance with the experimental sample as illustrated in Figure 5a. From the AFM measurements, we extracted a thickness of 25 nm for the MXene nanosheets. The thermal conductivity of ITO (*k*_ITO_ = 4 W/(m∙K)) and the perovskite (*k*_perovskite_ = 0.26 W/(m∙K)) are taken from the literature, while the information on the glass substrate is taken from the COMSOL database [65, 66]. To simulate the heating of the layer, we implemented a heat source on the MXene and the Perovskite layers. The power of the heat source is equal to the absorbed power during the measurements. The absorbed power on the MXene nanosheet (α = 78%) on top of the perovskite and on an area without the nanosheet (α = 19%) were measured within the Raman setup. As the glass substrate and the ITO are transparent, we estimate that most of the power is absorbed by the perovskite layer (α_perovskite_ = 19 ± 3%). Based on that, we extracted the absorptance of the MXene layer (α_MXene_ = 73 ± 10%). We set the incident laser power in the COMSOL model to 1 mW and the absorbed power was calculated through the corresponding absorbance values. The laser power was applied using a Gaussian beam function. For that purpose, we estimated the laser spot radius (ω_0_ = 500 nm) using the knife‐edge experiment method [67]. Then we calculated the temperature increase around the laser spot area within our model and fitted it to the experimental values by adjusting the relevant heat spreading characteristics. Hereby, the thermal boundary conductance between the MXene and the perovskite only has a weak impact on the results in comparison to the thermal conductivity. Therefore, we swept the thermal conductivity of the MXene and fitted the parameter at which the experimental and computational values fit.

Figure 5b shows the temperature distribution along the laser spot area with the fitted *k*_MXene_ parameter. Figure 5c shows the calculated temperature as a function of the thermal conductivity with an obtained fitting at *k*_MXene_ = 46 ± 22 W/(m∙K). From the results of the samples without perovskite, we fitted *k*_MXene_ = 59 ± 14 W/(m∙K), which is a reasonable match taking the uncertainties into account. In comparison to the other components in the system, especially the perovskite, the reported thermal conductivity of the MXene represents a particularly high value.

To study the improved heat‐dissipating properties of the MXene layer, we studied the temperature distribution in applicable ITO/MXene/perovskite structures. The geometry of this structure is indicated in Figure 5d. Figure 5e,f shows the temperature distribution observed in the sample without and with the presence of MXene. In this case, the heating source is applied only to the perovskite layer. Based on our previous calculation of the MXene thermal conductivity, we can estimate a reduction of the perovskite temperature of 20 ± 6% in the device where the MXene structure is employed, in comparison to the sample without MXene. Our results demonstrate that the functionalized MXene functions as an efficient heat‐dissipating layer between the ITO and perovskite layer. Although it is not directly inferred that this thermal heat dissipation is related to the observed operational stability of solar cells, it could synergistically relate this property to the observed enhancement in the long‐term stability observed for the MXene‐based PSCs under continuous light irradiation (ISOS‐L‐2) at high temperature [40, 68]. The enhanced high‐temperature operational stability originates from the combined effects of interfacial stabilization and improved thermal transport. The MXene:MeO‐2PACz interface reduces buried‐interface defects, improves charge extraction and carrier mobility, decreases lattice strain, and suppresses ionic accumulation. In parallel, Raman thermometry and finite‐element modeling demonstrate that the functionalized MXene nanosheets provide an efficient heat‐spreading pathway, resulting in a calculated reduction of approximately 20 ± 6% in the perovskite temperature. Therefore, heat dissipation should be regarded as a distinct and complementary contribution to the stability enhancement rather than its sole or quantitatively predominant origin [68].

## 3 Conclusions

In summary, we demonstrated that the application of the MXene: MeO‐2PACz interface layer applied as an HTL in iPSCs substantially improves the long‐term stability of the solar cells due to the heat‐dissipating properties of the MXene. These findings were obtained via stability assessment of the PSCs under continuous light irradiation conditions at high temperatures (ISOS‐L‐2) and under outdoor conditions (ISOS‐O‐2). We found that this novel interfacial passivation enhances the thermal stability of solar cells due to effective heat dissipation by MXene nanosheets through the study of the power and temperature dependence of Raman peak shifts. The COMSOL simulations showed that MXenes can reduce the perovskite temperature by roughly 20%. In addition, the modified devices show improved trap state reduction, energy band alignment, and charge extraction, and increased carrier mobility by means of CELIV measurements. The enhanced operational stability results from a synergistic mechanism. Raman thermometry and finite‐element modeling provide direct evidence that the functionalized MXene layer spreads heat and reduces the simulated perovskite temperature by 20 ± 6% [40]. In parallel, structural and optoelectronic measurements demonstrate improved crystallinity, reduced buried‐interface trap density, enhanced charge extraction, and suppressed ionic accumulation. We hope our work sheds light on the heat‐dissipating properties of MXenes and other 2D materials already employed in high‐temperature stability assessments of PSCs.

## Author Contributions

**Masoud Karimipour**: conceptualization, investigation, methodology, data curation, resources, formal analysis, validation, writing – original draft, writing – review and editing. **Timm Swoboda**: investigation, formal analysis, data curation, writing – original draft, writing – review and editing. **Kenedy Tabah Tanko**: formal analysis, writing – review and editing. **Javier Rodríguez‐viejo**: resources, investigation, writing – review and editing, validation. **Tiankai Zhang**: formal analysis, data curation, validation, writing – review and editing. **Maria Chiara Spadaro**: formal analysis, data curation, writing – review and editing. **Nil Monros Oliveras**: formal analysis, investigation, writing – review and editing. **Feng Gao**: resources, data curation, writing – review and editing. **Francesco Salutari**: investigation, formal analysis, writing – original draft, writing – review and editing. **Marianna Sledzinska**: investigation, writing – review and editing. **Jordi Arbiol**: writing – review and editing, validation, resources. **Monica Lira‐cantu**: methodology, supervision, writing – original draft, writing – review and editing, project administration, validation, funding acquisition. **Zhenchuan Tian**: investigation, formal analysis, writing – review and editing.

## Conflicts of Interest

The authors declare no conflicts of interest.

## Supporting information




**Supporting File**: advs77661‐sup‐0001‐SuppMat.docx.

## Data Availability

The data that support the findings of this study are available from the corresponding author upon reasonable request.
