# Supplementary material for: Improving High‐Temperature Operational Stability of Inverted Perovskite Solar Cells Through MXene Heat‐Dissipating Layers
Source: Adv Sci (Weinh). 2026 Sep 27:e77661. Online ahead of print. doi: 10.1002/advs.77661 (PMC13616322; doi:10.1002/advs.77661)
Supplement: Supplementary file 1 [file ADVS-9999-e77661-s001.docx]

Supporting Information

Improving High-Temperature Operational Stability of Inverted Perovskite Solar Cells through MXene Heat-Dissipating Layers

Masoud Karimipour^a, *^, Nil Monros Oliveras^a^, Zhenchuan Tian^a^, Timm Swoboda^a^, Francesco Salutari^a^, Marianna Sledzinska^a^, Maria Chiara Spadaro^a,c^, Kenedy Tabah Tanko^a^, Javier Rodríguez-Viejo^a,d^, Jordi Arbiol^a,b^, Tiankai Zhang^e^, Feng Gao^e^, and Monica Lira-Cantu ^a,*^

^a^ Catalan Institute of Nanoscience and Nanotechnology (ICN2), CSIC and BIST, Campus UAB, Bellaterra, 08193 Barcelona, Catalonia, Spain

^b^ ICREA, Pg. Lluís Companys 23, 08010 Barcelona, Catalonia, Spain

^c^ Department of Physics and Astronomy ‘Ettore Majorana', University of Catania, and CNR-IMM, Via S. Sofia 64, Catania, 95123 Italy

^d^ Departament de Fisica, Facultat de Ciencies, Universitat Autonoma de Barcelona (UAB), Bellaterra, Barcelona 08193, Spain

^e^ Department of Physics, Chemistry and Biology. (IFM), Linköping University, Linköping, 58183, Sweden.

E-mail: masoud.karimipour@icn2.cat, monica.lira@icn2.cat

[**1.	EXPERIMENTAL PROCEDURES	3**](#_Toc236757699)

[**1.1.	Materials	3**](#_Toc236757700)

[**1.2.**	**Delamination process of Ti_3_C_2_-T_X_ nanosheets and their functionalization with MeO-2PACz (MXene:MeO-2PACz nano flakes)**	3](#_Toc236757701)

[**1.3.**	**Preparation of perovskite, ETL and HTL precursor solutions**	3](#_Toc236757702)

[**1.4.	Device fabrication	4**](#_Toc236757703)

[**2.	Characterization Tools	4**](#_Toc236757704)

[**2.1.	Optical, electrical and structural characterizations	4**](#_Toc236757705)

[**2.2.**	**STEM/ EELS measurements**	6](#_Toc236757706)

[**2.3.**	**Trap density of states (tDOS) measurements by thermal admittance spectroscopy (TAS).**	6](#_Toc236757707)

[**2.4.**	**Indoor stability measurements**	7](#_Toc236757708)

[**2.5.**	**Electrical Characterizations of solar cells**	7](#_Toc236757709)

[**2.6.**	**Thermal conductivity measurements**	8](#_Toc236757710)

[2.7.	Device encapsulation for outdoor stability tracking tests	8](#_Toc236757711)

[**3.**	**Device characterizations & analysis after ISOS-L-2 assessment**	9](#_Toc236757712)

[**4.	Supporting Tables:	12**](#_Toc236757713)

[**5.	Supplementary Figures	16**](#_Toc236757714)

# 1. EXPERIMENTAL PROCEDURES

# 1.1.  Materials

Lead(Ⅱ) bromide (PbBr_2_, 99.99%), Lead(Ⅱ) iodide (PbI_2_, 99.99%), Cesium iodide (CsI_2_, 99.99%), Rubidium iodide (RbI_2_, 99.99%), formamidinium iodide (FAI), methylammonium iodide (MAI), and the hole transport layer material MeO-2PACz ((2-(3,6-Dimethoxy-9H-carbazol-9-yl)ethyl) phosphonic acid, and Bathocorpine(BCP), phenyl-C61-butyric acid methyl ester (PC_61_BM) as electron transport layer were acquired from Tokyo Chemical Industry (TCI) Co. Ltd. Dimethyl sulfoxide (DMSO), N, N-dimethylformamide (DMF), chlorobenzene (CB), anhydrous isopropanol (IPA), and LiCl from Merck Chemicals Co. Ltd. Tetrapropylammonium hydroxide (TPAOH), were obtained from Sigma-Aldrich. Ti_3_C_2_T_X_ nanosheet powder was supplied by Nanochemazone Inc. All materials were used as received unless specified otherwise.

1.2. **Delamination process of Ti_3_C_2_-T_X_ nanosheets and their functionalization with MeO-2PACz (MXene:MeO-2PACz nano flakes)**

Blending 15 mg Ti_3_C_2_T_X_ powder with 5 mL of TPAOH and 5mg of LiCl and stirring with vortex at 10000 rpm at ambient temperature for 12 hours, following that the supernatant was separated from the precipitation with centrifuging at 1000 rpm for 2 min and it was then washed with IPA and centrifuging three times, re-dispersed in 3 mL of IPA to acquire the dark green color solution as shown in Figure S1 and stored in the refrigerator for future use. A specific quantity (1 mg and 2 mg) of 2-(3,6-Dimethoxy-9H-carbazol-9-yl)ethyl)phosphonic acid (MeO-2PACz) were added to 2 mL of previous delaminated Ti_3_C_2_T_X_ solution and treated by bath ultrasonication for 20 min. The resulting MXene:MeO-2PACz dispersion was spin-coated at 2500 rpm for 20 s and annealed at 85 °C for 3 min.

1.3. **Preparation of perovskite, ETL and HTL precursor solutions**

Cs_0.05_(FA_0.85_MA_0.15_)_0.95_Pb(I_0.9_Br_0.1_)_3_ precursor solution was prepared by firstly, PbI_2_/PbBr_2_ precursor solution was prepared by dissolving 548.6 mg/ 57.06 mg of PbI_2_/PbBr_2_ in 1mL of DMF:DMSO (4:1 volume ratio) at 150ºC for 5-6 min, then allowed to cool down. Thereafter, it was injected to a vial containing 27.02 mg of CsI, 178.94 mg of FAI, and 17.41 mg of MABr and was shaken for 20 sec. and dissolved completely at 75ºC for 15 minutes to form the triple-cation perovskite Cs_0.08_MA_0.12_FA_0.8_Pb(I_0.88_Br_0.12_)_3_. For H3pp bulk passivation (H3pp:HP), 4.5 µl of H3pp stock solution (20 mg/ 0.5mL DMF) was injected into 0.5 mL of triple-cation perovskite solution and was shaken for 20 sec. 3 mg of MeO-2PACz was dissolved in 1mL of IPA and heated at 70 ^o^C to obtain a clear solution and used as HTL solution.

PC_61_BM was used as ETL material, therefor 20 mg of this material purchased from TCI was dissolved in 1 ml Chlorobenzene and heated at 55 °C for 20 min to dissolve it completely. 1mg of Bathocorpine (BCP) as buffer layer was dissolved in 1ml of IPA and heated up to 80 °C to obtain a clear solution.

# 1.4.  Device fabrication

Patterned ITO (6-8 Ω/sq, 2.5 cm×1.5 cm) coated onto glass substrates were washed with 10% Hellmanex solution firstly, then cleaned by bath ultrasonication with 2% Hellmanex solution, acetone, and isopropanol for 30 min, 10 min, 15 min subsequently. Afterwards, the substrates were treated with UV/Ozone for 20 min and transferred to the glovebox (Temperature: 18-20 ℃, O2: 3-6 ppm, H_2_O: 1-1.5 ppm) for solar cell fabrication immediately. MeO-2PACz layer as HTL was deposited using the 3mg/ml MeO-2PACz solution spin coating at 3000 rpm for 30 s and annealing at 100 ^o^C for 5 min. After cooling down to ambient temperature, The MXene:MeO-2PACz functionalized solution was spin coated at 2500 rpm for 20 s and annealed at 85 ^o^C for 3min. The resultant device is nominated as Modified in the manuscript. The perovskite films were fabricated by one-step deposition. Firstly, 50 μL 3-cation perovskite precursor solution was spin coated onto the substrates at 2000 rpm for 10 s and 5000 rpm for 30 s, chlorobenzene (CB) was used as anti-solvent in the last 15 s, then the film was annealed at 110 °C for 45 min. The resultant device is recalled as **Modified** in the entire manuscript**.** Thereafter, the electron transport layer was deposited onto the perovskite layer using PCBM solution with 1500 rpm, 30 sec and subsequently BCP was deposited at 4000 rpm for 20s and annealing at 90 ^o^C for 5 min. Finally, 100 nm Ag electrodes were evaporated thermally on the top of BCP by high vacuum PVD system with pressure of 8×10^-7^ atm for 2 h.

# 2. Characterization Tools

# 2.1.  Optical, electrical and structural characterizations

Current Density-Voltage (J–V) measurements were performed using a HelioSim-CL60 solar simulator from Voss electronic GmbH with a source meter (Keithley 2400) in the ambient atmosphere under 1 sun illumination (AM 1.5 G) at room temperature. 0.160 cm^2^ aperture area metal mask was used to define the active area. Reverse scans from 1.2 V to 0 V in all devices were set to a scan speed of 100 mV/s. Incident photon to current conversion efficiency (IPCE) were measured by Bentham PVE300 photovoltaic characterisation system, and aid in the use of the system and the control software, BenWin+, A monochromatic probe source Bentham TM300, 300mm focal length monochromator, with range 300-1200 nm, is assembled from a dual Xenon/quartz Halogen light sources. The output from the 75W Xenon lamp (300-700 nm) and Halogen (700-1200 nm) are coupled to the monochromator via an off-axis parabolic reflector. Photoluminescence (PL) spectra of the samples have been obtained by a PicoQuant system that offers both excitation and emission wavelength scans. The sample can be excited by a pulsed laser, LED, or Xe-flash lamp. The morphology of the products has been examined using a field emission scanning electron microscope (FEI Quanta 650 FEG ESEM), which is suitable for all types of samples (conductive, insulating, and wet materials). The applied acceleration voltage was 10 kV, and the base pressure was 1.5×10^-5^ Torr. The instrument has three detection systems: for secondary electrons, for back-scattered electrons, and for transmitted electrons. In addition, Energy-Dispersive X-Ray Spectroscopy (EDS) analysis is possible with this instrument, using the Inca 250 SSD XMax20 detector. This technique enables the chemical analysis of the sample via Energy-Dispersive X-Ray Analysis (EDX), where chemical maps and point analyses can be performed, getting to examine the chemical composition of a certain area of study in the sample. To perform cross SEM-EDS elemental mapping, from the champion Control solar cell after stability assessment, the device was placed on a stage in 13 mm from electron gun at voltage of 15 kV, and the image was first drift corrected and then elemental mapping was conducted. The crystal structural properties of the products have been characterized by XRD with the Malvern PANalytical X’pert Pro Materials Powder Diffractometer (MPD), which analyses at room temperature. The X-ray source is a ceramic tube emitting Cu Kα radiation, where λ = 1,540 Å. The geometry of the instrument follows the reflection Bragg-Brentano configuration, in which the sample is kept horizontal, and the system is θ-θ, with a vertical goniometer. The Malvern PANalytical X’pert Pro Materials Research Diffractometer (MRD) has also been used at room temperature. The X-ray source is the same as in the MPD, and the configuration of the device consists of a ω-2θ system with a horizontal goniometer, following the four-circle geometry. Fourier transformed infrared (FTIR) measurements were performed by using PMA50 Tensor 27. Ultraviolet photoelectron spectroscopy (UPS) and X-ray photoelectron spectroscopy (XPS) were performed using a SPECS Phoibos 150 hemispherical energy analyzer. The spectra were calibrated to the Carbon 1s orbital at 284.8 eV, and Al Kα X-ray source for XPS and He source (21.2 eV) for UPS, with a beam width of 100 μm, equipped with auto-neutralization, provided a total energy resolution of 2.9 meV. The contact angle of the films was evaluated using a Drop Shape Analyzer (KRÜSS DSA25S). A precise volume of pure water, 0.937 μL (±0.186 μL), was utilized for the measurements. The test was conducted under controlled conditions, with an average temperature of approximately 20 °C in ambient air.

2.2. **STEM/ EELS measurements**

Scanning transmission electron microscopy (STEM) experiments were conducted using a Thermo Fisher Scientific Spectra 300 operating at an accelerated voltage of 300 kV. EELS experiments were carried out using a Gatan Continuum equipped with a K3 direct electron detector. The energy spread of the zero-loss peak (ZLP) was measured at ~1 eV full-width at half-maximum (FWHM). The dispersion was 0.18 eV/ch, and a convergence angle of 22.7 mrad, camera length of 190 mm and spectrometer aperture diameter of 1 mm were used. Automatic ZLP alignment was enabled to correct the energy shift during the acquisition time in dual EELS mode. To minimize electron beam damage, EELS experiments were per-formed in the low-current condition; the screen current was less than 100 pA and the pixel time for acquisition was 10 ms.

2.3. **Trap density of states (tDOS) measurements by thermal admittance spectroscopy (TAS).**

Temperature-dependent capacitance measurements were carried out in a temperature-controlled sample holder^[4]^. Capacitance-frequency measurements were carried out in a frequency range of 20 Hz to 2 MHz in logarithmic steps using an Agilent E4980A LCR meter. The capacitance-voltage curve was obtained by measuring the capacitance as the applied D.C. bias voltage was scanned from -0.5 to 1.2 V. Based on the capacitance spectra measured at different temperatures, the trap density (*N*_T_) distribution in energy (*E*_ω_) was calculated with the following relations:

$N_{T}(E_{\omega})=-\frac{{´V}_{bi}}{qW}\frac{dC}{d\omega}\frac{\omega}{k_{B}T}$	(1)

$E_{\omega}=k_{B}Tln(\frac{\omega_{0}}{\omega})=k_{B}Tln\left( \frac{2\pi v_{0}T^{2}}{\omega} \right)$	(2)

where *V*_bi_ is the built-in potential and *W* is the depletion width (*V*_bi_ and *W* are derived from capacitance–voltage measurements); *C* is the capacitance measured at an angular frequency *ω* and temperature *T*; *k*_B_ is Boltzmann’s constant and *ω*_0_ is the attempt-to-escape frequency at temperature *T*; and *ν*_0_ is a temperature-independent constant, which can be obtained by fitting the relation of characteristic frequency with different T based on Equation 2.

2.4. **Indoor stability measurements**

by means of maximum power point tracking (MPPT) were performed with 6 min intervals using a Litos Lite (Fluxim AG) with constant N_2_ gas flow and connected to a cooling system at fixed temperature of **65 ^o^C** under simulated 1-sun using calibrated LED Wavelabs package.

2.5. **Electrical Characterizations of solar cells**

Electrical measurements were carried out using the PAIOS setup purchased from FLUXIM AG equipped with a LED lamp calibrated to 1 sun intensity. All the electrical measurements were done under constant N_2_ gas flow. Electrical impedance spectroscopy (EIS) at dark was performed for bias voltages ranging from 0.2 V to 1 V. with varying frequency from 1Hz to 2 MHz. Moreover, EIS at V_oc_ was measured at light intensities varying from 10% to 100% of the LED’s power. For Mott-Schottky analysis, capacitance was measured 10 kHz with varying voltages from -0.1 to 1.4 volts. Transient photo-current (TPC) measurements were carried out at light-pulse length of 500 μs with settling and follow up times of 500 μs and 200 μs respectively at 10% of light intensity modulation. For open circuit voltage decay (OCVD) measurements light-pulse length of 30 ms along with settling and follow up times of 10 ms and 15 ms were chosen respectively with 10% of light intensity pulses. Finally, the equivalent circuit fittings of Nyquist plots were performed using ZView software. Charge Extraction Linearly increasing voltage (CELIV) was performed with sweeping the voltage from 0 to 1.2, and the ramping rate was changed from 0.005 V/μs to 5 V/μs, with the injection time of 100 μs. Then the mobility of the carriers was calculated based on current injection across the swept voltages.

2.6. **Thermal conductivity measurements**

The Raman measurements were conducted by using a Horiba T64000 Raman spectrometer equipped with a single grating configuration of 2400 g/L. The system utilizes a 532 nm green laser (Cobolt) as both the probing and heating source. The laser was focused onto the sample with a high numerical aperture (NA = 0.5) and a magnification of 50x, achieving a laser spot size of approximately 500 nm. The spot size was measured using the Knife-edge experiment. The high-resolution capability of the spectrometer allows for the detection of spectral shifts with a resolution of approximately 0.05 cm^-1^. In the first set of experiments, the sample was initially heated by increasing the laser power. As a result of the temperature rise a red shift in the peak positions was observed. Subsequently, the material was externally cooled and heated using a variable-temperature cryostat (Linkam), from -40 °C to 40 °C, which was evacuated to a pressure of less than 5×10^−3^ mbar. The peak position of each spectrum was obtained by using a Lorentzian fitting function. The Raman maps were obtained by moving the position of the automatized sample stage with a Märzhäuser controller.

# 2.7.  Device encapsulation for outdoor stability tracking tests

*Device encapsulation:* Each substrate was sandwiched between two pieces of 60 mm × 60 mm × 3 mm glass (Climavit, S.A MX-M2651). Firstly, PSCs on substrates measuring 25 mm × 15 mm × 2.2 mm were placed, centralized on the bottom glass. Copper tapes were then glued with conductive silver epoxy to the cathodes and anodes of the PSCs. The glue was cured at 50 °C for 45 min. A butyl rubber edge seal with added desiccant (Quanex SET LP03, 3967) was applied as a frame on the glass, 0.5 cm from its edges. The copper tapes were laid between the bottom and middle butyl edge seal, followed by a top edge seal on the cover glass. Each edge seal was 1.7 mm thick. The package was laminated in a vacuum laminator (Meier Solar Solution, ICOLAM 10/08) at 100 °C. The vacuum of the chamber was evacuated to the minimum during 2 min. Then, the pressure applied on the package was gradually increased for 2 min up to 1000 mbar and maintained at 1000 mbar for 15 min before the pressure was released and the chamber was brought back to atmospheric pressure in 1 minute (total lamination time, 20 minutes). The samples were finally allowed to air cool outside the laminator.

*Outdoor stability*: Outdoor stability (ISOS-O-2) tests were performed in Barcelona, Spain (41.5°N, 2.1°E, Csa climate). PSCs were mounted on a dual axis sun tracker and performance was measured at MPP using an MP0205M24, LPVO maximum power point tracker. Solar intensity was measured with ISO 9060 / IEC 61724 Class A pyranometers while temperature and humidity were recorded using RK330-01 Atmospheric Temperature, Humidity & Pressure Sensor. All sensors were mounted on the same platform as the PSCs.

3. **Device characterizations & analysis after ISOS-L-2 assessment**

To further study the effect of this passivation strategy on the ion migration suppression, and any of its correlation to the spanning of stability behaviour of inverted solar cells, cross SEM images of two champion cells (PCE over 23% for both) were prepared after ISOS-L-2 assessment as shown in Figure S10a. It clearly shows that the Control device has degraded as indicated by a dashed yellow ellipse with a brighter contrast color compared to the rest of perovskite layer. It has also detached from the ITO/SAM layer which indicates the degradation has occurred from this side of device. To ensure this part of device degradation, an elemental mapping EDS analysis was performed by focusing the beam in this region of the device and the resultant contrasting images has been shown in Figure S10b. The In elemental mapping image has been plotted to ensure there has been no shift or drift in image during recording of EDS elemental mapping as its line is exactly matching the substrate line in electron image. Iodide and Cs images are showing clearly a deficit color/faded color with darker contrast compare to the rest of perovskite layer in the degraded region as indicated by ellipse in Figure S10a. It indicates that there has been a compositional change of 3-cation perovskite, that could be due to the segregation of PbI_2_, as this part has less Iodine concentration compared to the perovskite with higher stoichiometry amount of Iodine. To shed more light about the effect of ion migration on carrier lifetime and charge extraction, we performed series of EIS for the champion devices, before and after undergoing ISOS-L-2 protocol as shown individually in Figure S11 for each device. Figure S12a, show the Nyquist plots of the devices before (solid symbol) and after (open symbol) MPP tracking for 1000 h, at 65 ^o^C. Figure S12a show that before examining the stability protocol, the semicircle of the IS of the modified devices in 10%, and 20% of 1-Sun light intensity exposure, have higher resistance than the Control devices. Moreover, with the increase of light intensity to 100 %, the semicircle value for the modified device is smaller than that of Control. This observation is indicating that the MXene:MeO-2PACz interface modification is actually suppressing the recombination which is the dominant factor in low light intensities, where there is not a high rate of carrier generation ^1^, therefore the semicircle could be attributed to the recombination resistance. While in full sun intensity, where the high rate of carrier generation is dominant, the dominant process for the same configuration of the devices is charge extraction at the interfaces, thus the device with lower semicircle is the one with higher charge transfer resistance ^1, 2^. Figure S11 also show the individual evolution of Nyquist plots with light intensity variation, that the device resistance evolution for each case is gradually changing from 10% to 100% light intensity. In addition, the impedance evolution of the devices was investigated after performing ISOS-L-2 protocol. The plots with open symbols, show that after the stability assessment, in all light intensities, the Control device shows higher semicircle compared to Modified device. It is worth noticing that the Modified device has degraded 23%, and the Control performance has decreased almost 50%, of their initial values, thus their semicircle, is showing a reduction behavior compared to their counterparts before the ISOS protocol. This could be due to the increase of recombination that lowers the resistance part of semicircle. Moreover, the modified device in all light intensities as shown in Figure S11, and also In Figure S12a, shows lower values of cell resistance compared to the Control. This is actually because of high rate of degradation in the Control device compared to the Modified, that causes higher rate of recombination and decrease the charge transfer rate and consequently increases the charge transfer resistance. In addition, the imaginary component of impedance in high frequency (HF) region has increased significantly for the Control device compared to the Modified one and its maximum shifted to lower frequencies. This indicates higher chemical capacitance ^1^, which may be related to perovskite degradation and ionic segregation at the interfaces ^3^. To analyze these interpretations, we have performed a simulation using equivalent circuit suggested in Figure S11. This circuit suggests generally two different parts of high frequency (HF) and low frequency (LF) regions, that are attributed to all processed involving carrier extractions and ion mobilities, respectively. Figure S12b show the lifetime of HF and LF regions versus different light intensities, obtained from the simulations. In HF regime, the lifetime of carriers is longer for the Modified device compared to the Control device in both before and after the ISOS-L protocol. More interestingly, the lifetime in LF, has changed dramatically after the protocol assessment. For the control sample, τ_LF_ has decreased highly, while for the Modified device is increasing significantly. This behaviour can be observed directly from the Nyquist plots, as shown in Figure S11 and Figure c-f, open symbol plots, that the LF impedance behaviour of modified device is almost linear or could be considered as a semicircle with a very large radius, therefore it shows a very large resistance for Ions that is another clear indication of Ion migration suppression at the interface of HTL/perovskite in analogy with our previous results ^4^.

In conclusion we have shown extensively that this molecular intercalation of very thin MXene nanosheets can be utilized at the interface of HTL/perovskite layer to enhance crystallinity of inverted perovskite solar cells, reduce trap densities at the interface, and improve charge extraction together with immobilizing Ions at the interface to prohibit their interaction with ITO/SAM and suppress device degradation even at elevated temperatures.

# 4. Supporting Tables:

**Table S1:** Calculated Fermi level and Valence band positions of bare ITO, ITO/MeO-2PACz, ITO/MeO-2PACz with bare MXene deposited on top of it, and ITO/MeO-2PACz modified with MXene:MeO-2PACz nanosheets from Figure S7a&b.

| Sample type | Fermi level (E_f_) (eV) Vs Vac | Valence band (eV) Vs E_f_ | Valence band (eV) Vs Vac |
| --- | --- | --- | --- |
| Bare ITO | -4.15 | -3.16 | -7.31 |
| ITO/SAM(MeO-2PACz)  (Control) | -4.80 | -0.49 | -5.29 |
| ITO/SAM/pure MXene | -5.15 | -0.51 | -5.66 |
| ITO/SAM/MXene:MeO-2PACz  (Modified) | -4.86 | -0.67 | -5.53 |

**Table S2:** Comparison of PSCs applying MXenes and their stability assessment under different ISOS protocols. Tx = Time at which the PCE of the PSC reaches X % of the initial PCE value during a stability assessment. T_80_ is the most common value employed, for comparison purposes, when assessing stability of PSCs. T_80_= Time at which the PCE of the PSC reaches 80 % of the initial PCE value during a stability assessment. ISOS-D: In the Dark (no light irradiation), ISOS-T: Under Temperature (no light irradiation), ISOS-L: Under constant light irradiation, ISOS-O: Outdoor (variable light irradiation, temperature, light/dark cycling)

| Device type | Perovskite Absorber | ETL | HTL | MX**^*^** Role | ISOS protocol | T_80_ , Temp. | Ref |
| --- | --- | --- | --- | --- | --- | --- | --- |
| N  I | CsFaPbI_3_ | C60/BCP:**MX** | ITO/NiO_x_ | ETL | L-2 | 2300h,**63º±1.5** ºC | ^5^ |
|  |  |  |  |  | T-1 | 1080, 80 ºC |  |
| I | Cs_0.17_FA_0.83_Pb(I_0.83_Br_0.17_)_3_ | PCBM/BCP:**MX** | FTO/SAM | ETL | L-2 | 90h, 65ºC | ^6^ |
| N | CsFAMAPb(I_0.9_Br_0.1_)_3_/**MX** | ITO/SnO_2_ | Spiro-OMeTAD | Absorber | T-1 | 500h, 85ºC | ^7^ |
|  |  |  |  |  | L-1 | 350h, 25ºC |  |
| N | FA_0.85_MA_0.15_Pb(I_0.95_Br_0.05_)_3_ | ITO/SnO_2_:**MX** | Spiro-OMeTAD | ETL | D-3 | 1002h, 85 ^o^C | ^8^ |
| I | CsPbI_3_ | CPTA/BCP:**MX** | ITO/PTAA | ETL | L-2 | 1000h, 85ºC | ^9^ |
| I | FA_0.85_MA_0.1_Cs_0.05_PbI_3_ | PCBM/BCP | ITO/A-Ti_3_C_2_T_x_/4-PhCz | HTL | T-1 | 500 h (T_83.68_), 85 ^o^C | ^10^ |
| I | Cs_0.08_MA_0.12_FA_0.8_Pb(I_0.88_Br_0.12_)_3_ | PCBM/BCP | ITO/MeO-2PACz/  MX:MeO-PACz | HTL | L-2 | 687h, 65ºC | This study |
|  |  |  |  |  | O-2 | 1400 h (T_95_) |  |

*MX denotes to MXene, I: Inverted configuration; N: Normal configuration

| Type of perovskite | Device structure | ISOS-L-2 | Ref. |
| --- | --- | --- | --- |
| CsFaPbI_3_ | ITO/NiOx/HP/C60/BCP/MXene/Cu | T_80_= 2300h (65ºC) | ^5^ |
| Cs_0.17_FA_0.83_Pb(I_0.83_Br_0.17_)_3_ | FTO/SAM/HP/PMMA:MXene/PCBM/BCP/Ag | T_80_= 90h (65ºC) | ^6^ |
| Cs_0.05_Rb_0.04_(FA_0.85_MA_0.15_)_0.91_Pb(I_0.91_Br_0.09_)_3_ | ITO/PTAA/ HP/InSe/PC_70_BM/BCP/Ag | T_70_= 250h (65ºC) | ^11^ |
| Rb_0.05_Cs_0.05_MA_0.05_FA_0.85_Pb(I_0.95_Br0_.05_)_3_ | ITO/SAM:FC3283/HP/C_60_/BCP/Ag | T_90_= 180h (85ºC) | ^12^ |
| Cs_0.2_FA_0.8_PbI_3-x_Cl_x_ | ITO/NiO/TPATC/CsFAPbI_3-x_Cl_x_/C60/BCP/Cu. | T_92_= 1000h (63ºC) | ^13^ |
| Cs_0.05_(FA_0.88_MA_0.12_)_0.95_PbI_3_ | ITO /PTAA/AF/HP/AF/C_60_ / BCP/ Ag | T_90_= 1000h (85ºC) | ^14^ |
| Cs_0.05_(FA_0.98_MA_0.02_)_0.95_Pb(I_0.98_Br_0.02_)_3_ | ITO/NiO_x_/BT-T/HP/PCBM/BCP/Ag | T_95_= 1960h (65ºC) | ^15^ |
| Cs_0.05_(FA_0.95_MA_0.05_)_0.95_(PbI_0.98_Br_0.02_)_3_ | FTO/NiO_x_/2PACz+3-UA/HP/PCBM/BCP/Ag | T_91.2_= 1500h (65ºC) | ^16^ |
| Cs_0.05_FA_0.85_MA_0.10_PbI_2.95_Cl_0.05_. | ITO/NiO/Co-SAM/HP/PEAI/C_60_/BCP/Cu | T_87.5_= 672h (65ºC) | ^17^ |
| (FA_0.95_MA_0.05_)_0.95_Cs_0.05_Pb(I_0.95_Br_0.05_)_3_ | ITO/3-PA/HP/PEACI/ C_60_/BCP/Ag | T_86_= 1000h (ISOS-L-1, 25ºC) | ^18^ |
| FAPbI_3_ | ITO/NiO/Me-4PACz/HP-GSH/PEABr /PCBM/BCP/Au | T_78.44_= 336h (65ºC) | ^19^ |
| FACsPbI_3_ | FTO/Me-4PACz/HP-5-MH/ C_60_/BCP/Ag | T_92_= 2000h (85ºC) | ^20^ |
| FACsPbI_3_ | FTO/Me-4PACz/HP-1-MH/ C_60_/BCP/Ag | T_80_< 400h (85ºC) | ^20^ |

**Table S3:** Comparison of stability of perovskite solar cells using ISOS-L-2 protocol with different passivation and fabrication strategies.

**Table S4**: Parameter of the COMSOL simulation.

| **Parameter** | **Value** | **Meaning** |
| --- | --- | --- |
| **α_MXene_** | 73 % | Absorptance MXene |
| **α_perovskite_** | 19 % | Absorptance Perovskite |
| **d_Mx_** | 25 nm | Thickness MXene |
| **d_p_** | 150 nm | Thickness perovskite |
| **d_glass_** | 2 mm | Thickness glass |
| **d_ITO_** | 150 nm | Thickness ITO |
| **Δ*T*_Raman_** | $\Delta T\cdot P_{abs}$[K] | Expected Temperature |
| **Δ*T*** | 219 K/mW | Experimental temperature slope in Mxene in the ITO/perovskite/MXene:MeO-2PACz structure |
| **k_MX_** | Sweep parameter | Thermal conductivity MXene |
| **k_pe_** | 0.26 W/(m∙K) | Thermal conductivity perovskite |
| **k_ITO_** | 4 W/(m∙K) | Thermal conductivity of ITO |
| **P_MX_** | $PA\cdot\exp(-2\cdot\left( \frac{r^{2}}{w_{0}^{2}} \right))$ [W/m^3^] | Power profile of MXene heat source |
| **P_0_** | $P_{in}\cdot\alpha_{MXene}$ [W] | Absorbed Power MXene |
| **PA** | $\frac{2\cdot P_{0}}{\pi\cdot w_{0}^{2}\cdot d_{MX}}\text{[W/m}\text{3}\text{]}$ | Gaussian Power amplitude MXene |
| **P_p_** | $PAS\cdot\exp(-2\cdot\left( \frac{r^{2}}{w_{0}^{2}} \right))$ [W/m^3^] | Power profile of perovskite heat source |
| **PAS** | $\frac{{2\cdot P}_{0p}}{\pi\cdot w_{0}^{2}\cdot d_{pe}}\text{[W/m}\text{3}\text{]}$ | Gaussian Power amplitude perovskite |
| **P_0p_** | $P_{in}\cdot\alpha_{perovskite}\cdot(1-\alpha_{MXene}$)[W] | Absorbed Power perovskite |
| **P_in_** | 1 mW | Incident power |
| **r_0_** | 500 µm | Model length |
| **T_amb** | 299.15 K | Ambient temperature |
| **w0** | 0.5 µm | 1/e^2^ Laser spot radius |

# 5. Supplementary Figures

**Figure S1.** Bulk MXenes delamination process for the synthesis of MXene nanoflakes

**Figure S2.** (a) XRD and (b) HR-TEM images, SAD pattern and EDX spectrum of the corresponding power spectrum of the MXene nano flakes.

**Figure S3.** SEM images of highly flat MXene:MeO-2PACz nanosheets deposited on ITO/SAM substrates

**Figure S4.** Energy Dispersive Spectrum (a) and the corresponding elemental mapping of a flat large MXene: MeO-2PACz nanoflake on ITO/SAM film.

**Figure S5**: a) AFM topography images (left) of different MXene: MeO-2Pacz nanosheets deposited on ITO substrate, and their corresponding Roughness values across each nanosheet, b) 3D topography view of MXene: MeO-2Pacz nanosheets deposited on ITO substrate indicating total roughness of 16 nm across the film.

**Figure S6**: a)XRD of Halide perovskite films with and without modification, b) χ-scan XRD of devices for (001) Bragg peak, c) Strain plot for Chi-scan analysis of (220) Bragg peak of perovskite on ITO/SAM (Control: black square) and ITO/SAM/MXene:MeO-2PACz (Modified: blue triangle) substrates, d) Williamson-Hall analysis for calculation of microstrain of the perovskite films on the two different substrates.

**Figure S7**: a) work function (WF) & b) Valence band (VB) position measurements of bare ITO, ITO/SAM (MeO-2PACz), ITO/SAM with bare MXene deposited on top of it, and ITO/SAM modified with MXene:MeO-2PACz nanosheets, c) work function (WF) & d) Valence band (VB) position measurements of the perovskite film deposited on the unmodified and modified ITO/SAM substrates obtained from UPS analyses for the calculation of band diagram of the devices.

b)

**Figure S8:** Optimization of MeO-2PACZ deposition as HTL layer on ITO substrates for achieving highest PCE and stability as Control device. This step was performed since MeO-2PACz was dissolved in IPA as solvent.

(b)

(a)

**Figure S9.** a) Schematical illustration of ISOS-L-2 protocol set up for applying simultaneously thermal heating and simulated 1-Sun and performing MPP tracking for 1000 h, b) Humidity, temperature and input power tracking(W/m^2^) of outdoor measurements for ISOS-O-2 stability protocol experiment.

**Figure S10**: a) Cross SEM images of the selected champion devices of each category after performing ISOS-L-2 stability assessment. b) cross SEM image and EDS-elemental mapping of champion Control device after ISOS-L-2 protocol assessment, for In, Ag, Pb, Cs, I elements.

**Figure S11**: Equivalent circuit proposed for the simulation of impedance spectroscopy measurements using Z-view software, and independent Nyquist plots of the champion devices before (top) and after (bottom) ISOS-L-2 protocol assessment at different light intensities of simulated 1-sun.

**Figure S12:** a) Nyquist plots of champion devices before (solid) and after (open) performing ISOS-L-2 stability protocol measured at their open circuit voltages under 10, 20 50 and 100 % of simulated 1-Sun intensity, b) High frequency (HF), and low frequency(LF) life times of the devices obtained from simulated impedance spectroscopy measurements at different light intensities.

**References:**

1.	Karimipour, M.; Khazraei, S.; Kim, B. J.; Boschloo, G.; Johansson, E. M. J., Efficiency and Stability Enhancement of Perovskite Solar Cells Utilizing a Thiol Ligand and MoS2 (100) Nanosheet Surface Modification. *ACS Applied Energy Materials* **2021,** *4* (12), 14080-14092.

2.	Weber, S. A. L.; Hermes, I. M.; Turren-Cruz, S.-H.; Gort, C.; Bergmann, V. W.; Gilson, L.; Hagfeldt, A.; Graetzel, M.; Tress, W.; Berger, R., How the formation of interfacial charge causes hysteresis in perovskite solar cells. *Energy & Environmental Science* **2018,** *11* (9), 2404-2413.

3.	Chen, X.; Shirai, Y.; Yanagida, M.; Miyano, K., Effect of Light and Voltage on Electrochemical Impedance Spectroscopy of Perovskite Solar Cells: An Empirical Approach Based on Modified Randles Circuit. *The Journal of Physical Chemistry C* **2019,** *123* (7), 3968-3978.

4.	Karimipour, M.; Paingott Parambil, A.; Tabah Tanko, K.; Zhang, T.; Gao, F.; Lira-Cantu, M., Functionalized MXene/Halide Perovskite Heterojunctions for Perovskite Solar Cells Stable Under Real Outdoor Conditions. *Advanced Energy Materials* **2023,** *13* (44), 2301959.

5.	Yakusheva, A.; Saranin, D.; Muratov, D.; Gostishchev, P.; Pazniak, H.; Di Vito, A.; Le, T. S.; Luchnikov, L.; Vasiliev, A.; Podgorny, D.; Kuznetsov, D.; Didenko, S.; Di Carlo, A., Photo Stabilization of p-i-n Perovskite Solar Cells with Bathocuproine: MXene. *Small* **2022,** *18* (37), e2201730.

6.	Assunção, J. P. F.; Lemos, H. G.; Rossato, J. H. H.; Nogueira, G. L.; Lima, J. V. M.; Fernandes, S. L.; Nishihora, R. K.; Fernandes, R. V.; Lourenço, S. A.; Bagnis, D.; Santos, S. F.; Graeff, C. F. O., Interface passivation with Ti3C2Tx-MXene doped PMMA film for highly efficient and stable inverted perovskite solar cells. *Journal of Materials Chemistry C* **2024,** *12* (2), 562-574.

7.	Wan, Z.; Wei, R.; Wang, Y.; Zeng, H.; Yin, H.; Azam, M.; Luo, J.; Jia, C., Multifunctional MXene for Thermal Management in Perovskite Solar Cells. *Nano-Micro Letters* **2025,** *18* (1), 18.

8.	He, J.; Hu, G.; Jiang, Y.; Zeng, S.; Niu, G.; Feng, G.; Liu, Z.; Yang, K.; Shao, C.; Zhao, Y.; Wang, F.; Li, Y.; Wang, J., Dual-Interface Engineering in Perovskite Solar Cells with 2D Carbides. *Angew Chem Int Ed Engl* **2023,** *62* (41), e202311865.

9.	Heo, J. H.; Zhang, F.; Park, J. K.; Joon Lee, H.; Lee, D. S.; Heo, S. J.; Luther, J. M.; Berry, J. J.; Zhu, K.; Im, S. H., Surface engineering with oxidized Ti3C2Tx MXene enables efficient and stable p-i-n-structured CsPbI3 perovskite solar cells. *Joule* **2022,** *6* (7), 1672-1688.

10.	Wei, X.; Yu, B.; Zhang, Y.; Cai, Q.; Ouyang, X.; Yu, H., Interface Engineering With Annealed MXene and SAM Molecules for High‐Performance Inverted Perovskite Solar Cells. *Advanced Energy Materials* **2026,** *16* (24).

11.	Tsikritzis, D.; Chatzimanolis, K.; Tzoganakis, N.; Rogdakis, K.; Zappia, M. I.; Martín-García, B.; Bagheri, A.; Beydaghi, H.; Děkanovský, L.; Sofer, Z.; Bellani, S.; Bonaccorso, F.; Kymakis, E., Engineering of the perovskite/electron-transporting layer interface with transition metal chalcogenides for improving the performance of inverted perovskite solar cells. *Sustainable Energy & Fuels* **2024,** *8* (10), 2180-2190.

12.	Zhang, X.; Wang, Y.; Zhang, K.; Tao, M.; Guo, H.; Guo, L.; Song, Z.; Wen, J.; Yang, Y.; Hou, Y.; Song, Y., Reinforcing Coverage of Self-assembled Monomolecular Layers for Inverted Perovskite Solar Cells with Efficiency of 25.70 %. **2025,** *64* (13), e202423827.

13.	Sukhorukova, P. K.; Ilicheva, E. A.; Gostishchev, P. A.; Luchnikov, L. O.; Tepliakova, M. M.; Balakirev, D. O.; Dyadishchev, I. V.; Vasilev, A. A.; Muratov, D. S.; Kiselev, D. A.; Ilina, T. S.; Luponosov, Y. N.; Di Carlo, A.; Saranin, D. S., Triphenylamine-based interlayer with carboxyl anchoring group for tuning of charge collection interface in stabilized p-i-n perovskite solar cells and modules. *Journal of Power Sources* **2024,** *604*, 234436.

14.	Xu, J.; Xi, J.; Dong, H.; Ahn, N.; Zhu, Z.; Chen, J.; Li, P.; Zhu, X.; Dai, J.; Hu, Z.; Jiao, B.; Hou, X.; Li, J.; Wu, Z., Impermeable inorganic “walls” sandwiching perovskite layer toward inverted and indoor photovoltaic devices. *Nano Energy* **2021,** *88*, 106286.

15.	Li, L.; Wei, M.; Carnevali, V.; Zeng, H.; Zeng, M.; Liu, R.; Lempesis, N.; Eickemeyer, F. T.; Luo, L.; Agosta, L.; Dankl, M.; Zakeeruddin, S. M.; Roethlisberger, U.; Gratzel, M.; Rong, Y.; Li, X., Buried-Interface Engineering Enables Efficient and 1960-Hour ISOS-L-2I Stable Inverted Perovskite Solar Cells. *Adv Mater* **2024,** *36* (13), e2303869.

16.	Zhang, Z.; Wu, T.; Qin, Z.; Chen, M.; Xiang, W.; Chen, Z.; Wang, Y.; Guo, Z.; Matsushima, T.; Han, L., Synergistic Improvement of Structural Ordering and Interface Binding of Hole Transport Monolayer for Efficient Inverted Perovskite Solar Cells. **2025,** *15* (27), 2500572.

17.	You, W.; Chang, W.; Xue, H.; Xiang, X.; Wang, Y.; Zhou, Y.; Zhou, J., Polar heterocyclic molecule-assisted co-assembly of monolayers at the NiOx/perovskite interface for high-efficiency and stable inverted perovskite solar cells. *Journal of Materials Chemistry A* **2026,** *14* (11), 6678-6687.

18.	Wei, K.; Wang, W.; Zhang, C.; Hu, J.; Sun, G.; Liu, D.; Pang, S.; Yang, L.; Tang, W.; Zhang, J. J. A. E. M., Multidentate Molecular Anchoring for Enhanced Interfacial Stability and Reliable Perovskite Solar Cells. **2026,** *16* (18), e70806.

19.	Jin, M.; Yang, D.; Shen, H.; Tang, S.; Liu, L.; Wang, Y.; Zheng, C.; Kuang, J.; Wang, C.; Naveed, F. J. A. M., Multifunctional Glutathione Enables ISOS‐Robust Inverted Perovskite Solar Cells via Dipole Engineering and Redox‐Driven Self‐Healing. **2026,** *38* (27), e73036.

20.	Huang, Z.; Liu, B.; Liang, Z.; Xu, H.; He, H.; Liu, W.; Zhou, H.; Zhang, Y.; Ye, J.; Pan, X. J. A. M., Positional Methylation Isomer Additives Regulate the Degradation Pathway of Perovskites. **2026,** *38* (20), e20432.
